# Slow Off-Rate Modified Aptamer (SOMAmer) Proteomic Analysis of Patient-Derived Malignant Glioma Identifies Distinct Cellular Proteomes

**DOI:** 10.3390/ijms22179566

**Published:** 2021-09-03

**Authors:** Thatchawan Thanasupawat, Aleksandra Glogowska, Christopher Pascoe, Sai Nivedita Krishnan, Maliha Munir, Farhana Begum, Jason Beiko, Jerry Krcek, Marc R. Del Bigio, Marshall Pitz, Yaoqing Shen, Victor Spicer, Kevin M. Coombs, John Wilkins, Sabine Hombach-Klonisch, Thomas Klonisch

**Affiliations:** 1 Department of Human Anatomy and Cell Science, University of Manitoba, Winnipeg, MB R3E 0J9, Canada; thatchawan.t@gmail.com (T.T.); Aleksandra.Glogowska@umanitoba.ca (A.G.); nive1996@live.com (S.N.K.); munirmn@myumanitoba.ca (M.M.); Farhana.Begum@umanitoba.ca (F.B.); jkrcek@shaw.ca (J.K.); marc.delbigio@umanitoba.ca (M.R.D.B.); mpitz@cancercare.mb.ca (M.P.); Sabine.Hombach-Klonisch@umanitoba.ca (S.H.-K.); 2 Department of Physiology & Pathophysiology, University of Manitoba, Winnipeg, MB R3E 0J9, Canada; CPascoe@chrim.ca; 3 Children’s Hospital Research Institute of Manitoba, Winnipeg, MB R3E 3P4, Canada; Kevin.Coombs@umanitoba.ca; 4 Department of Surgery, University of Manitoba, Winnipeg, MB R3E 0Z2, Canada; jbeiko@hsc.mb.ca; 5 Department of Pathology, University of Manitoba, Winnipeg, MB R3E 0Z2, Canada; 6 Department of Internal Medicine, University of Manitoba, Winnipeg, MB R3E 0Z2, Canada; John.Wilkins@umanitoba.ca; 7 Research Institute in Oncology and Hematology (RIOH), CancerCare Manitoba, Winnipeg, MB R3E 0V9, Canada; 8 Canada’s Michael Smith Genome Sciences Centre, BC Cancer, Vancouver, BC V5Z 1L3, Canada; yshen@bcgsc.ca; 9 Manitoba Centre for Proteomics and Systems Biology, Winnipeg, MB R3E 3P4, Canada; Victor.Spicer@umanitoba.ca; 10 Medical Microbiology & Infectious Diseases, Rady Faculty of Health Sciences, College of Medicine, University of Manitoba, Winnipeg, MB R3E 0J9, Canada

**Keywords:** glioma, glioblastoma, proteomic clusters, SOMAmers, patient cell isolates

## Abstract

Malignant gliomas derive from brain glial cells and represent >75% of primary brain tumors. This includes anaplastic astrocytoma (grade III; AS), the most common and fatal glioblastoma multiforme (grade IV; GBM), and oligodendroglioma (ODG). We have generated patient-derived AS, GBM, and ODG cell models to study disease mechanisms and test patient-centered therapeutic strategies. We have used an aptamer-based high-throughput SOMAscan^®^ 1.3K assay to determine the proteomic profiles of 1307 different analytes. SOMAscan^®^ proteomes of AS and GBM self-organized into closely adjacent proteomes which were clearly distinct from ODG proteomes. GBM self-organized into four proteomic clusters of which SOMAscan^®^ cluster 4 proteome predicted a highly inter-connected proteomic network. Several up- and down-regulated proteins relevant to glioma were successfully validated in GBM cell isolates across different SOMAscan^®^ clusters and in corresponding GBM tissues. Slow off-rate modified aptamer proteomics is an attractive analytical tool for rapid proteomic stratification of different malignant gliomas and identified cluster-specific SOMAscan^®^ signatures and functionalities in patient GBM cells.

## 1. Introduction

Malignant gliomas account for 78% of malignant primary brain tumors and include astrocytoma (AS) and oligodendroglioma (ODG), the oncogenic derivatives of the astrocytic and oligodendroglial lineages, respectively. AS constitute the largest population of malignant gliomas (>63%) and include highly proliferative and invasive anaplastic astrocytoma (grade III) and glioblastoma multiforme (grade IV; GBM). ODG account for 2–4% of all primary brain tumors and have a better prognosis than most other malignant gliomas. GBM is a rare tumor with an annual incidence of 5 to 8 per 100,000 of population but constitutes about 60% of all primary human brain tumors [1]. Despite surgical resection (debulking) or biopsy to the extent that is safe followed by radiation and chemotherapy [2,3], GBM has one of the worst 5-year survival rates among all human cancers [4,5]. Combined chemo-radiation treatment increases the median overall survival to about 14.6 months from 12.1 months with radiotherapy alone [6]. The addition of tumor treating fields may increase median survival to 20.9 months [7]. Current treatment options for malignant glioma remain limited and few patients achieve longer than 3-year survival [8].

Proteins execute cellular functions and account for the majority of oncology drug targets. Changes in protein composition and activity are major contributors to GBM progression, which includes proliferation and differentiation of GBM cells, their invasion into surrounding brain tissue, and the emergence of cellular mechanisms of therapeutic resistance. While large scale gene expression analysis of GBM identified distinct genetic subtypes and their molecular drivers [9,10,11], fewer proteomic studies have been performed on mostly smaller numbers of patient GBM tissues/cells or other brain tumors [12,13,14,15,16,17], with the exception of a most recent comparative proteogenomic and metabolomic analysis on 99 GBM tissue samples [18]. Currently, only a few proteins (and their mutated versions) are considered prognostic and predictive biomarkers for GBM and are used for prognosis stratification and selection of GBM patients for specific treatments [15,19,20,21,22].

High content quantitative proteomic analytical approaches with high sensitivity and specificity are anticipated to advance our understanding of the biology of glioma and help excel the discovery of new clinically relevant biomarkers and drug targets. SOMAscan^®^ 1.3K assay is a multiplexed proteomic analysis platform that uses Slow Off-rate Modified Aptamers (SOMAmers) for protein binding and enables the simultaneous relative quantification of 1307 different human proteins in each of up to 90 samples [23,24]. SOMAscan^®^ is highly specific and sensitive with a median lower limit of quantitation and detection at 40 pM and 100 fM, respectively [24]. The SOMAscan^®^ assay spans 8 Log_10_ of concentration with a median of 4.2 Log_10_ per SOMAmer, which is comparable to that achieved with antibody-based assays. Among many disease applications, this assay has been employed to identify proteomic signatures in non-small cell lung cancer [25], ovarian cancer [26,27], mesothelioma [28,29], and hepatocellular cancer [30].

We have applied SOMAscan^®^ proteomic technology to determine proteomic profiles of early passage cell culture samples isolated from fresh surgical specimens of brain tumor patients with GBM (*n* = 54), anaplastic astrocytoma (*n* = 13), and oligodendroglioma (*n* = 21). More than half of the >1300 proteins detected by the SOMAscan^®^ 1.3K assay are involved in inflammation and cellular signaling processes highly relevant to these malignant gliomas [23,31,32]. The SOMAscan^®^ proteomes confirmed an expected close relationship of GBM and AS, both being astrocytic in origin. AS and GBM proteomes were clearly distinct from ODG cellular proteomes. SOMAscan^®^ 1.3K segregated the 54 GBM cell isolates into four distinct GBM proteomic clusters. We successfully validated several protein candidates in patient GBM cells and corresponding GBM tissues. Bioinformatics analysis of the GBM SOMAscan^®^ proteomic clusters predicted biological networks with different complexity. SOMAscan^®^ technology is an attractive tool for high-throughput proteomic characterization of primary patient glioma cell isolates.

## 2. Results

### 2.1. Malignant Glioma Pathologies Have Distinct SOMAscan^®^ Cellular Proteomes

**Figure 1:**
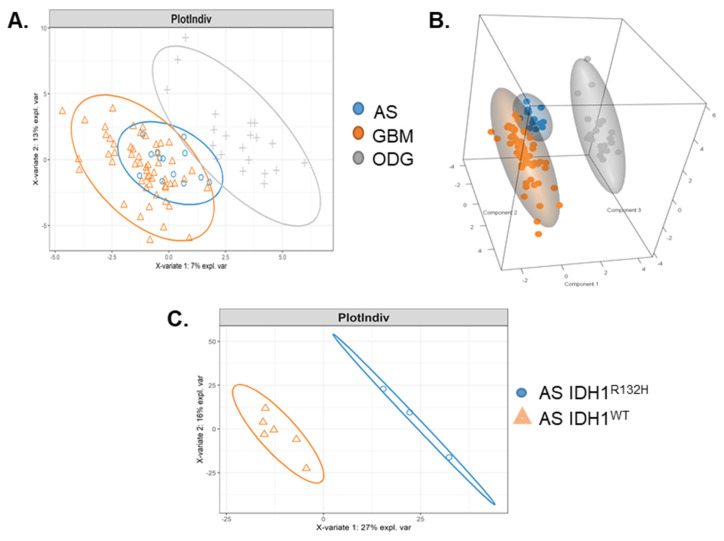
(**A**) Two-dimensional (component 1 and component 2) and (**B**) three-dimensional (components 1–3) clustering of tumor cells based on their proteome by sparse partial least squares discriminant analysis (sPLS-DA). Each point represents a sample, ellipse represents 95% confidence interval. Astrocytoma (AS; blue). Glioblastoma (GBM; orange). Oligodendroglioma (ODG; grey). (**C**) Two-dimensional clustering by sPLS-DA of AS cells with clinically diagnosed IDH1^WT^ (orange) and IDH1^R132H^ (blue) mutation showed distinct SOMAscan 1.3K proteomes for AS with IDH1^R132H^ mutation. The numbers on the axis indicate how much of the variation between points can be determined by the proteins that make up each component. The proteins on the x-axis and the y-axis contribute to 27% and 16% of the variability between the groups, respectively. The points mostly separate along the left and right direction (x-axis) which means that those proteins are likely to be different between the groups.

A total of 88 samples of patient-derived cell isolates at early passages (1–3) from three confirmed malignant glioma pathologies (54 glioblastoma (GBM), 13 anaplastic astrocytoma (AS), 21 oligodendroglioma (ODG)) underwent SOMAscan^®^ 1.3K proteomic analysis. Sparse Partial Least Squares Discriminant Analysis (PLSDA) revealed three distinct cellular proteomic profiles corresponding to the three malignant glioma pathologies as shown in 2D plots (Figure 1A) and 3D spatial representation (Figure 1B). PLSDA performed on a total of nine AS cell isolates with either isocitrate dehydrogenase 1 (IDH1) wildtype (IDH1^WT^; *n* = 6) and IDH1^R132H^ mutant (*n* = 3) revealed distinct SOMAscan^®^ proteomes of anaplastic AS with IDH1^R132H^ mutant (Figure 1C). The number of components and variables per component to use was determined through a tuning procedure, in line with the mixOmics protocol recommendation [33]. Three components with 21, 10, and 20 variables (components 1–3) enabled a clear separation of the three glioma types. Area under the curve from ROC (receiver operating characteristic) curves using the three components and selected variables were AS vs. others: 0.95, GBM vs. others: 0.98, ODG vs. others: 1. Common to all but one patient diagnosed with ODG, the loss of heterozygosity (LOH) of 1p36 and 19q13 chromosomal regions was confirmed by FISH analysis (data not shown). Clinical data for all glioma cases are summarized in Table 1A–C. Clinical pathology tests for immunoreactive glial fibrillary acidic protein (GFAP) on tissues had been performed in 16/54 cases (30%) of GBM, 11/13 cases (85%) of AS, and 17/21 cases (81%) of ODG (data not shown). For the six GBM cell isolates tested, we confirmed the clinical GFAP immunostaining results (Supplementary Material Figure S1).

### 2.2. Patient GBM Cell Isolates Segregate into Four SOMAscan^®^ Proteomic Clusters

**Figure 2:**
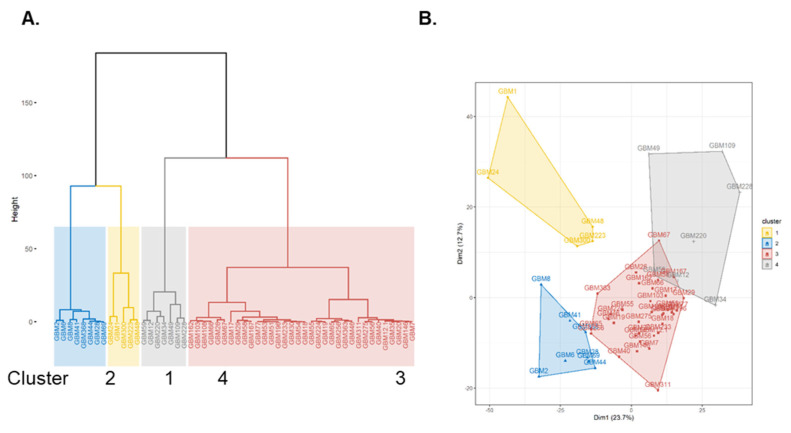
Principal Component Analysis (PCA) and cluster analysis of patient GBM cells. (**A**) Hierarchical clustering of GBM by similarity of their proteomes. (**B**) Hierarchical clusters plotted on PCA showing clusters in dimensions 1 and 2.

**Figure 3:**
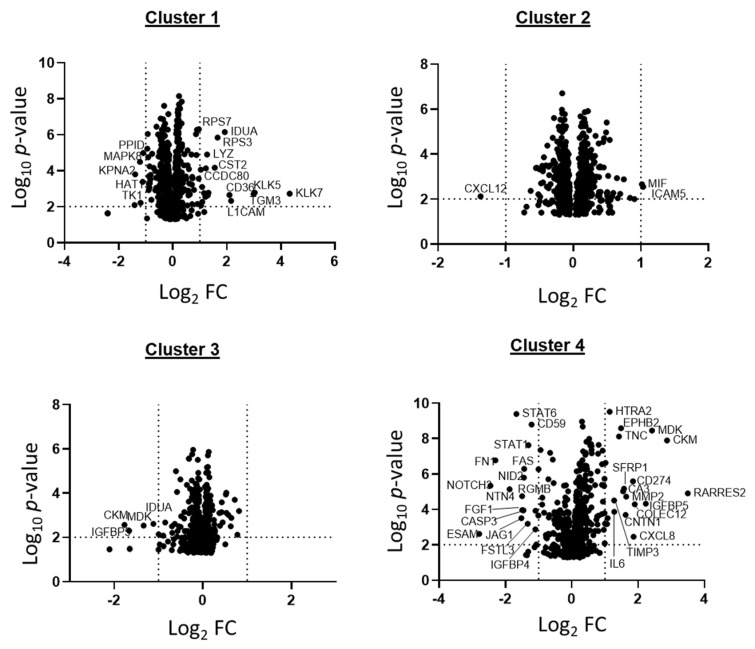
Volcano plot of GBM proteomic clusters. Volcano plot showing the relative protein contribution to the proteomic cluster 1–4 observed with SomaScan. Labelled proteins had fold changes ≥2 (log2 FC ≥ 1) and *p*-values of ≤0.01.

SOMAscan^®^ 1.3K assay identified four distinct proteomic signatures, referred to as clusters 1–4. Proteomic cluster affiliations of the GBM SOMAscan^®^ data were determined by PCA followed by hierarchical clustering on the first 3 principal components from the PCA and partial least squares discriminate analysis (PLS-DA) (Figure 2A,B). Of all patient GBM cell isolates (*n* = 54), cluster 3 contained the largest number with 63% of all cases (*n* = 34), followed by clusters 2, 4, and 1 with 15% (*n* = 8), 13% (*n* = 7) and 9% (*n* = 5), respectively (Figure 2A,B). PCA data for proteomic clusters 3 and 4 overlapped, indicating a closer relationship between these two clusters (Figure 2B). Clinical data revealed that 5 of 7 GBM patients of cluster 4 (71%) had survival times of less than 9 months and were all males (Table 1A). Additionally, cluster 4 included a recurrence (GBM-109) from a female patient where we had also collected cells from her primary GBM (GBM-54) which was grouped in cluster 3. The cluster specific distribution of all Somalogic protein analytes is shown as volcano plots and proteins with fold change (FC) ≤ or ≥2 (log_2_ FC ≤ or ≥1) and *p*-values of ≤0.01 are highlighted (Figure 3). The highest number of significantly regulated proteins were observed in cluster 4 (*n* = 31) followed by cluster 1 (*n* = 16), whereas in clusters 2 and 3 only three and four analytes met the significance thresholds, respectively (Figure 3).

### 2.3. SOMAscan^®^ Proteomic Clusters Are Validated in GBM Cells and Corresponding Tissues

**Figure 4:**
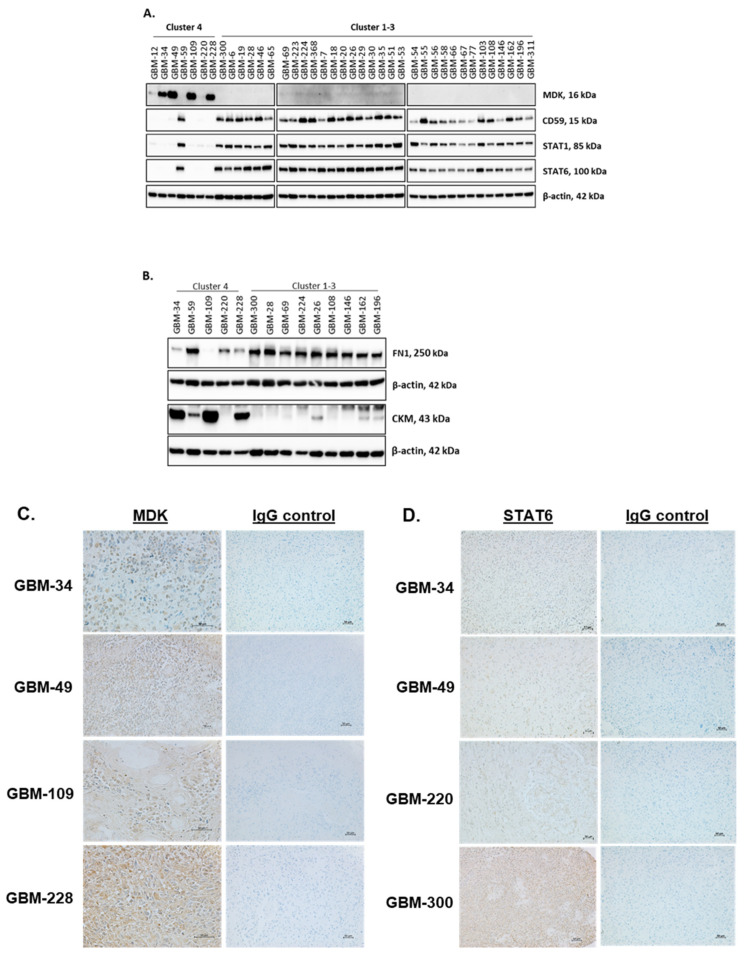
Validation of GBM protein targets. Representative Western blot validation of cluster 4 protein targets (**A**) MDK, CD59, STAT1, and STAT6 and (**B**) FN1 and CKM in representative GBM cell isolates of proteomic clusters 1–4. β-actin was used as a loading control and Western blots were performed as independent duplicates. Immunohistochemical staining of patient GBM tissues corresponding to selected GBM cell isolates. (**C**) MDK immunostaining was exclusively observed in GBM tissues of corresponding GBM cells of cluster 4 that were immunopositive in Western blot analysis. (**D**) Negligible STAT6 immunoreactivity in GBM tissues of corresponding GBM cell isolates of cluster 4 that were devoid of STAT6 as shown by Western blots. In accordance with the Western blot results, STAT6 immunostaining was positive in corresponding tissue sections of cluster 1 member GBM-300. The results of the IgG negative control experiments are shown. Details on the antibodies used are listed in Table 2. Representative immunohistochemical images of at least three independent experiments are shown. Magnifications: ×50 and ×100.

SOMAscan^®^ results were validated for several proteins which were selected based on the significance criteria for both FC and *p*-values and the availability of suitable antibodies for immunodetection. This included validation of CKM (creatine kinase, isoform M) and MDK (midkine) which were up-regulated in cluster 4 but down-regulated in cluster 3, as well as FN1 (fibronectin 1; −1.70 log_2_ FC), STAT6 (−1.27 log_2_ FC), STAT1 (−0.84 log_2_ FC), and B-cell factor CD59 (−0.93 log_2_ FC) as significantly down-regulated proteins unique to cluster 4 (Figure 3). Using total cell lysates from all patient GBM cell isolates of cluster 4, GBM-300 of cluster 1, and GBM isolates randomly assigned from clusters 2 and 3, we successfully validated the SOMAscan^®^ results for protein candidates MDK, CKM, CD59, STAT1, STAT6, and FN1 by Western blot analysis run in duplicates with β-actin serving as loading control (Figure 4A,B). In agreement with the SOMAscan^®^ data and volcano plot results (Figure 3), GBM isolates of cluster 4 expressed MDK and CKM proteins, while those in clusters 1–3 had negligible amounts (Figure 4A,B). While consistently present in clusters 1–3, protein levels for CD59, STAT1, STAT6, and FN1 were negligible in cluster 4 GBM cellular proteomes, with the exception of cluster 4 member GBM-59 (Figure 4A,B).

**Figure 5:**
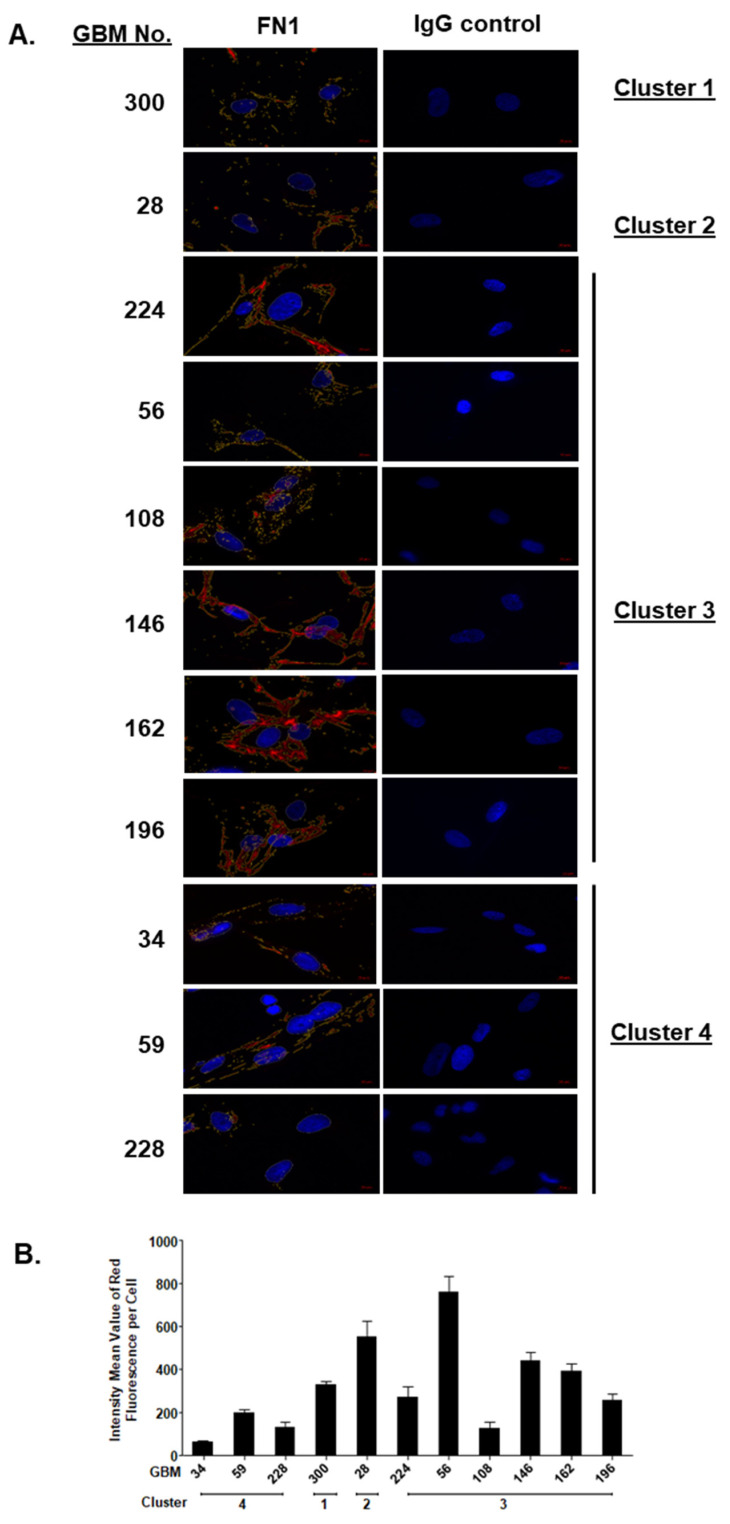
FN1 in patient GBM cell isolates. (**A**) Representative composite immunofluorescence images showing FN1 (red fluorescence) in patient GBM cell isolates (left) of different cluster affiliations (right) and corresponding IgG negative controls. All images shown were taken at identical exposure times for intensity analysis. Fibrous FN1 matrix assemblies were detected in GBM cells of clusters 1–3, whereas FN1 immunostaining weak and punctuate in GBM cells of cluster 4. The area for fluorescence intensity quantification is delineated with a yellow line. (**B**) Intensity analysis was performed on 30 GBM cells per patient and demonstrated reduced immunoreactivity for FN1 in cluster 4 GBM cells. These results confirmed the corresponding Western blot data. Representative immunohistochemical images of at least three independent experiments are shown.

We successfully validated the presence of MDK (Figure 4C) and absence of STAT6 proteins (Figure 4D) in corresponding patient tumor tissues of cluster 4 GBM members, with GBM tissues from cluster 1 member GB-300 serving as positive control for STAT6 (Figure 4C,D). Thus, the SOMAscan^®^ proteomes of early passage GBM cell isolates appeared to reflect the protein expression levels in GBM tissues. Additionally, we performed quantitative immunofluorescence densitometry to validate the down-regulated FN1 protein expression and its effect on FN1 matrix formation in GBM cells of the four clusters. We confirmed a significant and exclusive down-regulation of FN-1 (−1.70 log_2_ FC) protein in all but one (GBM-59) cluster 4 GBM cell lysates (Figure 4B). This coincided with weak granular FN1 matrix immunoreactivity, whereas GBM cells of proteomic clusters 1–3 produced a dense FN1 fibrillary matrix of higher mean fluorescence intensity (Figure 5A,B).

**Figure 6:**
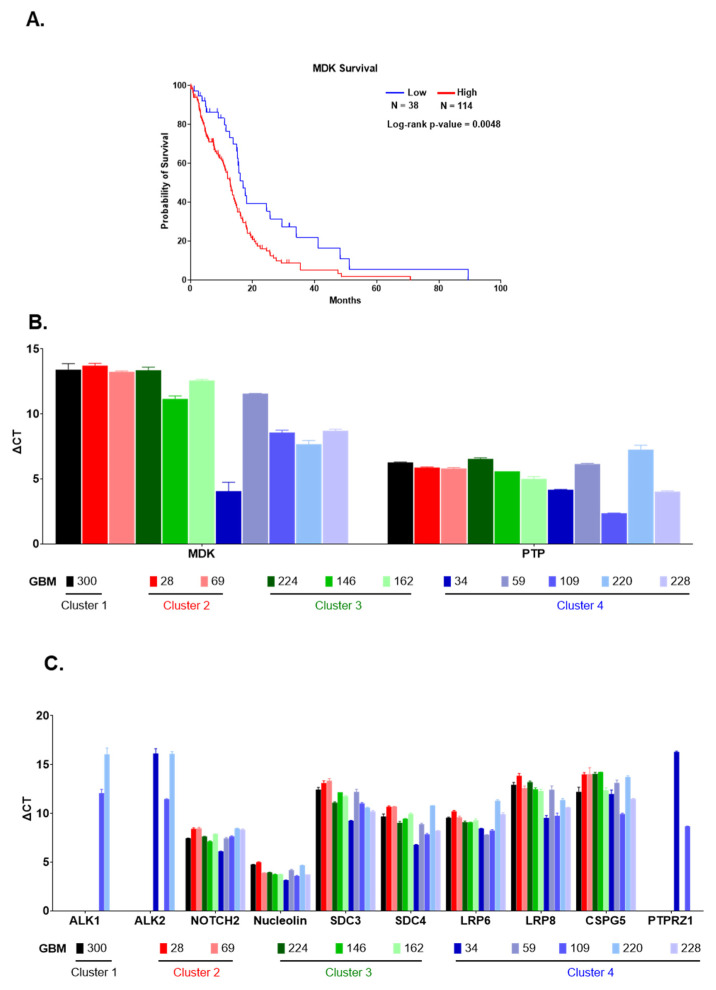
MDK family ligands and their receptors expression in patient GBM cells. (**A**) Kaplan Meier overall survival curve with log-rank test was used to determine the prognostic significance of MDK expression in GBM patients in the TCGA database. High MDK expression correlated with significantly reduced survival time of GBM patients. Q-PCR results are shown for the expression of (**B**) MDK and PTN and (**C**) 10 different putative MDK receptors in selected GBM cell isolates color-coded by clusters affiliation. Delta CT results are shown with higher expression levels denoted by the smaller columns.

Of all proteins identified by SOMAscan^®^ to be significantly altered in a cluster-specific manner, MDK was the only protein that qualified as a prognostic marker for poor outcome in GBM based on TCGA data (Figure 6A). Hence, we decided to investigate the MDK cytokine family of secreted heparin-binding growth factors in more detail. The gene activities of the two known MDK family ligands, MDK and the structurally and functionally related PTN (pleiotrophin; not captured by the SOMAscan^®^ 1.3K assay) were quantified by qPCR in patient GBM cells of different cluster affiliations (Figure 6B). In agreement with our Western blot data (Figure 4A), increased MDK transcripts levels were detected for cluster 4 members with high MDK protein levels, but lower in GBM-59 or GBM in clusters 1–3 with non-detectable MDK protein levels in Western blot (Figure 6B). All GBM cell isolates irrespective of cluster affiliation, expressed relatively high levels of PTN transcripts which suggested that the cluster-specific differences in MDK protein content were the result of differences in MDK transcriptional gene activity in these GBM (Figure 6B). Next, we used qPCR to analyze the transcriptional activity of putative MDK/ PTN receptor genes, namely ALK1, ALK2, NOTCH2, nucleolin, SDC3, SDC4, LRP6, LRP8, CSPG5, and PTPRZ1. With the exception of the recurrence GBM-109 in cluster 4, the expression of ALK1 and ALK2 was negligible in the GBM members of clusters 1–4 tested (Figure 6C). Varying levels of expression were observed for the other putative MDK receptors. There was a trend towards higher receptor expression levels in cluster 4 members but this was not statistically significant (Figure 6C).

### 2.4. Different Signaling Networks among SOMAscan^®^ GBM Proteomic Clusters

**Figure 7:**
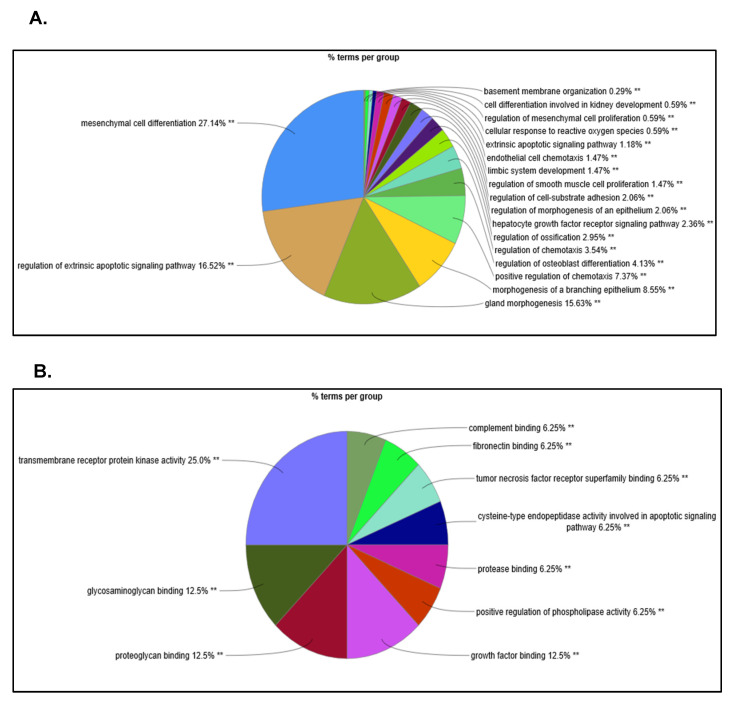

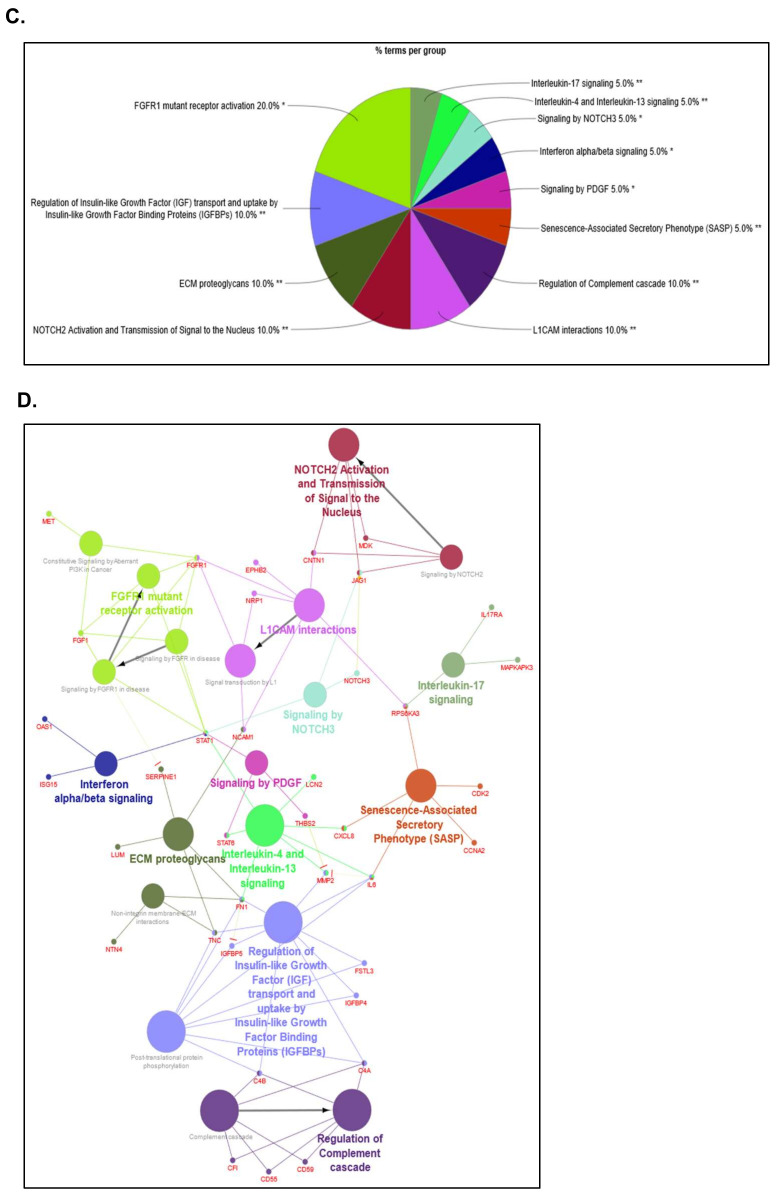
Bioinformatic analysis of proteomic cluster 4. Gene Ontology (GO) analysis of proteomic cluster 4. The pie chart shows groups of color-coded GO terms for (**A**) biological processes (**B**) and molecular functions based on proteomic data from GBM cell isolates of cluster 4. (**C**) The pie chart shows prevalent Reactome pathway terms enriched in proteomic cluster 4 GBM members. (**D**) Reactome pathway network of proteomic cluster 4 showing a signaling network of several inter-connected signaling nodes (color-coded), including major signaling hubs related to signaling factors IGF/IGFBPs, IL4/13/17, NOTCH2, and complement cascade. * and ** indicate the level of significance GO terms at *p* < 0.01 and *p* < 0.001, respectively.

We used Cytoscape (V3.8) with ClueGO plug-in (v2.5.7) to apply gene ontology (GO) methodology to all up- and down-regulated proteins of the 54 GBM SOMAscan^®^ proteomes to identify biological processes, cellular components, and molecular functions specific for the proteomic clusters in these GBM cells. The network complexity predicted by GO analysis was highest for proteomic cluster 4 (Figure 7A–D). SOMAscan^®^ identifying the highest number of proteins with significant differences in protein expression (>0.6 log_2_ FC; <−1 log_2_ FC) in cluster 4, followed by cluster 1. GO analysis of GBM cell proteomic cluster 4 discerned a total of 19 different biological processes of which the majority (92%) contributed to five major categories, including morphogenesis (34.2%), mesenchymal differentiation (27.7%), extrinsic apoptotic signaling (17.7%), and regulation of chemotaxis (12.4%) (Figure 7A). Proteomic cluster 4 supported several molecular functionalities (*n* = 10), with transmembrane ligand-receptor interactions (43.8%; transmembrane receptor protein kinase activity/growth factor binding/TNF receptor superfamily binding) and extracellular matrices proteins (31.3%; binding to proteoglycans, glycosaminoglycans, and fibronectin) accounting for 75% of these GO molecular functionalities (Figure 7B). Reactome pathway analysis of cluster 4 GBM cell proteomes revealed an interconnected signaling network composed of 12 pathways, each supported by at least three proteins from the SOMAscan^®^ 1.3K assay (Figure 7C,D). This included intercellular signaling via soluble (interleukins), membrane-anchored (Notch signaling) and extracellular matrix components (proteoglycans) as well as diverse intracellular signal transduction processes (e.g., PI3K, Notch, L1CAM) (Figure 7D). By comparison, network analysis identified only three solitary mainly transcriptional and cell cycle transition processes in cluster 1 proteomes (Supplementary Material Figure S2A–D) and none of the top up- and down-regulated proteins in proteomic clusters 2 and 3 met these significance criteria (Figure 3A–C). Corresponding GO analyses for clusters 2 and 3 only revealed basic biological processes and predicted involvement of few molecular functions for cluster 2 proteomes, while failing to specify any cellular components, molecular functions, or Reactome pathways with coherent connectivity for GBM proteomic cluster 3 (Supplementary Material Figure S3A–C).

## 3. Discussion

We have used a multiplexed aptamer-based SOMAscan^®^ 1.3K proteomic assay with simultaneous relative quantification of >1000 protein analytes for proteomic profiling of 89 patient-derived GBM, AS, and ODG malignant glioma cells. The SOMAscan^®^ 1.3K assay was developed as a high-throughput platform for the discovery of biomarkers and clinically relevant drugable proteins which is reflected in the high representation of secreted and membrane-associated proteins and a preference for analytes involved in inflammatory processes [24]. Despite the limited number of analytes interrogated, the resulting proteomes reflected the different origins of the gliomas. PCA and 3D sPLS-DA demonstrated similar but distinct proteomes for astrocytoma grade III (anaplastic AS) and grade IV (GBM). The proteomes of glioma of astrocytic origin (AS, GBM) segregated clearly from the proteomes of cell isolates derived from clinically diagnosed ODG patients. Intriguingly and despite a low number of AS isolates tested, we were able to identify SOMAscan^®^ 1.3K proteomes that were clearly distinct between anaplastic AS with IDH1^WT^ and IDH1^R132H^ mutation. Anaplastic AS with IDH1^R132H^ mutation have a more favorable prognosis [34,35,36]. The SOMAscan^®^ results demonstrated that the cultured IDH1^WT^ and IDH1^R132H^ AS cells retained the expression of distinct proteomes. Common to all-but-one ODG patient, but absent in astrocytic gliomas, was a loss of heterozygosity of 1p36 and 19q13 chromosomal regions in fluorescence in-situ hybridization [37].

SOMAscan^®^ reflected heterogeneity in proteomes among the GBM cell isolates. Tumor heterogeneity is pronounced in GBM and results from diverse regional histopathology, coexistence of different GBM subtypes and heterogeneity of GBM stem cell populations within each tumor [11,38,39]. Hierarchical clustering divided the GBM proteomes into four distinct clusters, with the most populated cluster 3 (*n* = 34) and cluster 4 (*n* = 7) showing partial overlap. This cluster relationship may, in part, explain the divergent validation results for GBM-59 (Figure 4A,B, Figure 5A,B and Figure 6B). Of all cluster 4 proteomes, the GBM-59 proteome showed greatest overlapped with proteomic cluster 3 (Figure 2B). SOMAscan^®^ proteomes from cells obtained from paired primary and recurrent GBM isolates demonstrated a transition from a cluster 3 (GBM-54) to a cluster 4 proteome (GBM-109) in the same GBM patient. Based on our GO analysis data, this reflected an evolution within approx. one year from a lower complexity primary GBM to a complex cluster 4 proteomic network in the recurrence. This concurs with recently reported progressive heterogeneity in transcriptomes and (phospho-) proteomes of primary and recurrent GBM, making predictions on clinical outcome and treatment challenging [12,40]. The recently released SOMAscan^®^ 8 k proteomic assay version is expected to provide a more detailed insight into cluster-specific cellular network dynamics and proteomic changes during GBM differentiation [41]. We anticipate that the 8K and future even larger SOMAscan^®^ assays are expected to accelerate proteomic analysis and become attractive tools in multi-omics platform initiatives [18].

We successfully validated six significant up- and down-regulated protein targets using cellular protein extracts, live cells, and corresponding FFPE tumor tissues obtained from the same GBM patients. Four of seven GBM cell isolates in cluster 4 strongly expressed MDK [42]. Unique among the top 20 up- and down-regulated proteins in SOMAscan^®^ GBM proteomes, the Human Protein Atlas identified high MDK expression as a predictive marker of poor prognosis in GBM (https://www.proteinatlas.org/ENSG00000110492-MDK/pathology/glioma, accessed on 5 January 2021). MDK is a secreted cytokine and heparin-binding factor that has been identified as a liquid biomarker in glioma and other tumors [43]. MDK is an important factor in the development and progression of high-grade astrocytoma and neuroblastoma [44,45,46,47] suggesting a role as tumor promoter in the brain. Secreted MDK promotes chronic inflammation and cellular immune responses in different pathologies, including neuropathologies [48,49], and is considered to be an attractive therapeutic target [50,51]. We excluded the possibility that the increased MDK expression in GBM cells may be due to culture conditions by identifying immunoreactive MDK expressed by GBM cells in corresponding patient GBM tissues. We concluded that the production of MDK protein was an inherent property of these GBM cells. The co-expression of MDK and the structurally related pleiotrophin (PTN), a heparin-binding brain mitogen not covered in the SOMAscan^®^ 1.3K assay, predicts short survival in GBM [52]. Irrespective of cluster affiliation or MDK protein content, all tested GBM cells consistently expressed PTN transcripts (Figure 6B, Table 1A). GBM-34, GBM-109, and GBM-228 had high MDK protein levels but matching high *MDK* transcript levels were only detected in GBM-34, whereas the other two cluster 4 members showed relatively low *MDK* transcriptional gene activity (Figure 4A and Figure 6B). The reasons for this discrepancy in GBM-109 and GBM-228 is likely complex. It is tempting to suggest that MDK protein levels in GBM cells are under the control of different molecular mechanisms shown to target both MDK RNA and protein. This includes RNA-binding protein HOW shown to enable mesoderm spreading during early fly embryogenesis by specifically down-regulating the Drosophila MDK and PTN homolog miple [53]. In addition, the ubiquitin–proteasomal system has been shown to regulate cellular MDK protein levels and functionality [54]. MDK and PTN interact with a plethora of surface receptors to initiate tumor promoting cell motility/invasion, survival, and drug resistance [55,56,57,58,59]. This includes protein tyrosine phosphatase ζ (PTPζ), anaplastic lymphoma kinase (ALK), syndecans-1, -3, and -4, integrins and low-density lipoprotein (LDL)-receptor-related proteins (LRP) 6 and 8 [60,61,62,63,64]. GBM cells from all four clusters expressed up to 10 different MDK/PTN receptor genes (Figure 6C), suggesting that these GBM cells can respond to MDK and PTN cytokines produced by the glioma microenvironment [65] and/or produced auto-/ paracrine by GBM cells, as demonstrated for GBM-34, -49, -109, and -228.

The metabolic enzyme creatine kinase, muscle isoform M (CKM), but not brain-type CKB, was a highly upregulated protein in cluster 4 GBM but only weakly or not expressed in GB members of clusters 1–3. Brain- and muscle-type CK isoforms were described in glial (astrocytes and Bergmann glia) and Purkinje neurons, respectively, of normal human brain [66] and a shift from CKB to increased M-isoform expression has been reported in high grade astrocytoma and GBM [67,68]. CK catalyses the reversible transphosphorylation between ATP and creatine to generate ADP and phosphocreatine. The complex of CK and high-energy product phosphocreatine (PCr) shuttles between ATP production sites (cytoplasmic glycolysis or mitochondrial oxidative phosphorylation) and subcellular locations of ATP consumption to serve as important temporal and spatial energy supplier for a plethora of ATP-dependent processes essential for cellular functions and survival [69]. Little is known about the regulation and functions of CKM in GBM. Our patient GBM cell models may be valuable new tools to address the role of endogenous CKM in GBM bioenergetics [70]. While the SOMAscan^®^ 1.3K assay detected both CKM and CKB, it does not include mitochondrial U-type CKMT1. Phospho-proteomic studies detected a specific down-regulation of CKMT1 isoform in the striatum of both MDK and PTN knockout mice [71]. We are investigating CKM as a potential new MDK target in GBM which may explain the concurrent high MDK and CKM protein levels in GBM-34, -109, and -228.

As predicted by the SOMAscan^®^ 1.3K assay, we successfully validated the GBM cluster-specific changes for CD59, FN1, STAT1, and STAT6 proteins in GBM cells and, for STAT6, also in corresponding GBM tissues. While this demonstrated the potential of this aptamer-based technology as a discovery tool for new biology and biomarkers, these assay results also pose new questions on the functional relevance and possible therapeutic implications of diminished protein levels of CD59, FN1, STAT1, and STAT6 proteins. The SOMAscan^®^ data may also reveal potential vulnerabilities of GBM cluster 4 members, with CD59 and FN1 serving as examples. CD59, in concert with membrane cofactor protein CD46 and decay accelerating factor CD55, facilitates resistance to complement mediated damage. Of the three factors, CD59 is critical for the protection of human U87 and U251 glioma cell lines and selected patient GBM cell lines from complement attack [72,73]. As for FN1, anaplastic astrocytoma and glioblastoma express this extracellular matrix protein at higher levels than low grade glioma [74,75,76]. The suppression of FN1 was shown to cause growth reduction, enhanced sensitivity to temozolomide and extend survival times of GBM xenografted mice [77,78]. Our patient GBM cell models offer alternative ways to study the effect of FN1 protein level and matrix deposition on FN1 functions in glioma signaling events that promote tumor proliferation, EMT, migration/tissue invasion/metastasis, survival, and treatment resistance [79].

## 4. Materials and Methods

### 4.1. GB Patient Tissue Samples and Cell Culture

GB patient tissues were provided from Winnipeg Health Sciences Center. Ethics protocol #H2010:116 was approved by the University of Manitoba and the Health Sciences Center Department of Pathology ethics boards and patient consent was obtained in all cases prior to tissue collection. For this study, we analyzed 54 glioblastoma (GBM), 13 anaplastic astrocytoma (AS), and 21 oligodendroglioma (ODG) cell isolates cultured in DME/F12 containing 10% FBS at 37 °C in a humidified 5% CO_2_ atmosphere. Clinical data of the tumor samples are summarized in Table 1A–C. Formalin fixed and paraffin embedded (FFPE) patient GBM tumor tissues corresponding to GBM cell isolates were used for validation studies.

### 4.2. Sample Preparation and SOMAscan^®^ Analysis

Protein extraction of patient GB cells at early passages (1–3) was done in M-PER lysis buffer (M-PER Mammalian Protein Extraction Reagent, Thermo Fisher, Ottawa, ON, Canada). Briefly, cell pellets were washed with PBS 3 times before incubating with M-PER lysis buffer with agitation for 5 min at room temperature (RT) and samples were centrifuged at 16,000× *g* to remove cell debris. Supernatants were collected and a BCA Protein Assay Kit (Thermo Fisher) was used to measure protein concentrations. All proteins samples were normalized to 75 µL at 200 µg/mL total protein concentration and stored at −80 °C prior to analysis. We used the 1.3K SomaLogic biomarker discovery assay (Somalogic, Boulder, CO, USA) composed of Slow Off-rate Modified Aptamer reagents (SOMAmers) that had been generated by Selected Evolution of Ligands by Exponential Enrichment (SELEX) to selectively bind a broad range of human proteins, with a preference for secreted proteins (47% secreted proteins, 28% extracellular epitopes, 25% intracellular proteins) [23,80]. These proteins detected by the SOMAmers belonged to a broad range of biological families, including cytokines, proteases, protease inhibitors, growth factors, hormones, cell surface receptors, kinases, and structural proteins. Sample preparation for the SOMAscan^®^ assay was performed according to the manufacturer’s instructions in 96-well plates with a semi-automatic Tecan Freedom Evo 200 high throughput system. The SOMAscan^®^ assays were run by SomaLogic. Briefly, protein samples were incubated with Cyanine-3 labelled SOMAmer reagents that had been immobilized onto streptavidin-coated beads via a biotin moiety linked to each SOMAmer by a photo-cleavable linker. Unbound and non-specifically bound proteins were removed from the beads by consecutive washes prior to protein conjugation with NHS-biotin reagent. After the labeling reaction and additional washes, proteins bound to SOMAmers and unbound SOMAmer reagents were released from the beads by cleaving the photo-cleavable linker with ultraviolet light. Beads were pelleted and the supernatant of photo-cleaved biotinylated protein bound to SOMAmers as well as unoccupied SOMAmers were incubated with a second set of streptavidin-coated beads to capture the biotin-labeled protein-SOMAmer complexes. Subsequent washes removed unoccupied SOMAmers before SOMAmer reagents were released from their cognate proteins using denaturing conditions. The unique sequence information of each SOMAmer reagent was utilized for hybridization-based custom DNA microarrays to quantify the DNA content using the fluorescence signal intensities of Cyanine-3 conjugated with the SOMAmers. The analysis, quality controls, calibrators, and criteria for the acceptance of assay data were determined by the manufacturer. Following data normalization and calibration and prior to any analysis, signal intensities expressed as relative fluorescent units (RFU) were log2 transformed to Soma expression values which were directly proportional to the amount of target analytes in the corresponding samples.

### 4.3. Sparse PLS-DA

To identify proteins important in the distinction between cells isolated from patients with GBM, AS, and ODG, we performed sparse partial least squares discriminate analysis (sPLS-DA) using the mixOmics package in R (version 6.10.9). sPLS-DA is well suited to performing both data reduction and variable selection in dataset where the number of variables outnumbers the number of samples [33,81]. The absolute value of the loading score for each variable indicates its importance in distinguishing the groups along that component. Variables were color coded based on the tumor group with the highest mean abundance. The sign (positive or negative) for the loading score indicates the direction of the eigenvalue from zero on the given component. The AUC (area under the curve) was calculated using the mixOmics package in R as part of the cross-validation process using one vs. all comparisons [82].

### 4.4. Hierarchical Clustering

We performed hierarchical clustering on the GBM SOMAscan^®^ data to identify cluster affiliations of GBM cells. Hierarchical clustering on principal components (HCPC) was performed using the FactoMineR package in R (version 2.3). We performed hierarchical clustering on the first three principal components from the principal component analysis (PCA). This function returned a list of proteins whose abundance values were used to discriminate the clusters. Significance was determined by testing the null hypothesis “the mean in the cluster is equivalent to the overall mean”, with a significance threshold set at *p* < 0.05.

### 4.5. Principal Component Analysis and Partial Least Squares Discriminate Analysis

We performed PCA on the SOMAscan^®^ data of 54 GBM samples to identify the spatial relationship between different GBM cell proteomic signatures. After hierarchical clustering, PLS-DA was performed to highlight the separation of GBM cell isolates with a proteomics signature of proteomic cluster 4 from the remaining GBM cell isolates in clusters 1 to 3. Analysis was performed in R using the stats package (version 3.6.1), factoextra (version 1.0.7), and mixOmics (version 6.10.9) packages. Volcano plots were generated to display proteins with significant changes in each cluster with a significance threshold set at FC ≥ ±2; *p*-value ≤ 0.05. The fold changes were calculated by taking the Log2 abundance of a protein in a given cluster and subtracting it from the average Log2 abundance in the other three clusters. This resulted in the Log2FC relative to the other three clusters. 

### 4.6. Western Blot Analysis

Proteins (10–20 µg/lane) were separated on 7.5% and 12% SDS-PAGE gels and transferred onto nitrocellulose membranes. Non-specific protein binding sites were blocked by incubation with 5% nonfat milk in Tris-buffered saline plus 0.1% Tween-20 (TBS/T) for 1h at RT. Primary antibodies were incubated overnight at 4 °C. Membranes were washed 3× with TBS/T before incubating with HRP-conjugated secondary antibodies for 2 h at RT. Specific binding was visualized with ECL Clarity (Bio-Rad, Mississauga, ON, Canada). All Western blots were performed using a Bio-Rad Laboratories system and ChemiDoc MP Gel documentation. All primary antibodies used for Western blots are listed in Table 2. ImageLab software version 6.1 (Bio-Rad) was used to quantify protein band intensities. Beta-actin was used as loading control and for normalization of protein bands.

### 4.7. Immunodetection of Proteomic Targets in Patient GBM Cells and Tissues

For immunofluorescence imaging, patient GBM cells were seeded onto APTES ([3aminopropyl] triethoxysilane) coverslips and fixed with 3.7% formaldehyde for 30 min at RT on the next day. Cells were permeabilized with Triton X-100 for 10 min, non-specific antibody binding sites were blocked for 1h and exposed to FN1 (fibronectin 1) antibody (Table 2) overnight at 4 °C. Cells were washed 3× in PBS and incubated with corresponding secondary antibodies for 1h at RT. Cells were counterstained using 1:60,000 DAPI for 5 min and coverslips were then mounted onto glass slides using Fluoromount G (ThermoFisher, Waltham, MA, USA). Images were taken with a Zeiss AXIO Imager.Z2 fluorescence microscope with an oil objective (×63) and ZEN imaging software. Quantification of FN1 immunofluorescence was performed on images taken at identical exposure times. FN1 immunofluorescence was quantified for 30 GBM cells for each patient isolate investigated using the Zen 3.0 pro image analysis module. Intensity threshold function was used to determine FN1 fluorescence intensity. Because of low FN1 immunofluorescence in GBM-34 and GBM-108 cells the intensity threshold was set to 150, whereas for the other nine patient GBM isolates the threshold was 350. For immunohistochemistry, deparaffinated human GBM tissue sections were incubated with 3% H2O2 in methanol for 20 min at RT in the dark to quench endogenous peroxidase. Antigen retrieval was performed by boiling the tissue sections in citrate buffer at pH 3.0 for 4 min and incubated at 90 °C for 30 min. Tissue sections were incubated with blocking buffer (10% goat normal serum in TBS/Tween-20) for 1 h at RT prior to incubation with MDK (Midkine) and STAT6 antibodies at 4 °C overnight (Table 2). Rabbit isotype IgG (Vector Laboratories, Burlington, ON, Canada) at the same concentration as the primary antibodies was used as negative controls. Sections were incubated with biotinylated IgG (1:200) (Vector Laboratories) for 1h at RT followed by incubation with avidin complexed to biotin-conjugated horseradish peroxidase (Vectastain Elite ABC kit; Vector Laboratories) for 30 min. Immunostaining was developed with DAB substrate (Thermo Scientific), sections were counterstained with hematoxylin and coverslipped for imaging with a bright field M2 microscope (Zeiss, Jena, Germany). 

### 4.8. RNA Isolation and Quantitative Reverse Transcriptase Polymerase Chain Reaction (qPCR)

Total RNA was collected for the qPCR detection of transcript expression levels of MDK, pleiotrophin (PTP), and several of their cognate receptors, including receptor-type tyrosine-protein phosphatase zeta (PTPRZ1), anaplastic lymphoma kinase ALK1 and ALK2, NOTCH2, nucleolin, syndecan (SDC) 3, SDC4, low-density-lipoprotein (LDL) receptor-related protein (LRP) 6 and LRP8, and chondroitin sulfate proteoglycan (CSPG) 5. Primers are listed in Table 3. The qPCR was performed with a QuantStudio^®^ 3 system (Applied Biosystems, Ottawa, ON, Canada). The delta C_T_ (ΔC_T_) method was used for data analysis using QuantStudio^®^ Design & Analysis software. Samples were normalized to the expression of GAPDH.

### 4.9. Bioinformatics Analysis

UniProt IDs and Entrez GeneIDs were used for network and pathway analyses. Cytoscape (version 3.8) with ClueGO V2.5.7 plug-in was used for Gene Ontology (GO) and Reactome pathway enrichment analyses (National Institute of General Medical Sciences, Bethesda, MA, USA) [83,84]. The ClueGO V2.5.7 plug-in generates functionally grouped GO annotation networks from a large cluster of genes. GO categories were divided into biological process, cellular component, and molecular function terms. *p*-values were calculated using the hypergeometric test and adjusted for multiple testing with Benjamini–Hochberg method. Adjusted *p*-values < 0.05 were considered statistically significant as denoted by ** *p* < 0.001, * *p* < 0.01, without star *p* < 0.05.

## 5. Conclusions

Slow off-rate modified aptamer-based high content quantitative SOMAscan^®^ multiplexed assay was successfully used for the proteomic stratification of novel patient-derived cell isolates collected and cultured from three different types of malignant gliomas. Using this proteomic strategy, patient-derived GBM cells segregated into four distinct proteomic clusters with different marker proteins and molecular networks. These novel patient-derived glioma cell models may aid in the identification of new molecular pathways and therapeutic responses in human glioma.

## Figures and Tables

**Table 1:** Clinical data of (**A**) 54 GBM samples. (**B**) 13 patients with anaplastic astrocytoma. (**C**) 21 patients with oligodendroglioma included in this study.

**A.**						
**No.**	**Sex**	**Age at Diagnosis**	**IDH1 Status**		**Survival (Months)**	**Proteomic Cluster**
1	f	57	ND		24.1	1
24	f	45	ND		8.9	
48	f	76	ND		20.7	
223	f	83	negative for IDH1 (R132H)		9.1	
300	f	34	negative for IDH1 (R132H)		18.6	
Median survival					16.3	
2	f	72	ND		18.5	2
6	f	63	ND		0.4	
8	m	78	ND		2.2	
28	f	45	ND		29.1	
41	m	72	ND		29.5	
44	m	63	ND		58.4	
69	m	49	mutant IDH1 (R132H)		67.6	
368	m	51	negative for IDH1 (R132H)		11.5	
Median survival					27.2	
7	f	34	ND		72.8	3
12.1	m	59	ND		86.9	
17	m	63	ND		2.8	
18	f	55	ND		6.9	
19	f	49	ND		19.3	
20	m	65	ND		3.1	
26	m	76	ND		7.9	
29	m	59	ND		10.7	
30	m	35	ND		9.2	
35	f	51	ND		20.8	
40	m	52	ND		30.9	
46	m	36	ND		54.5	
51	f	45	ND		9.7	
53	m	63	ND		1	
54	f	40	ND		26.1	
55	m	25	ND		10.7	
56	m	66	ND		7.9	
58	m	68	ND		7.5	
65	f	59	ND		19.4	
66	m	53	ND		6.2	
67	f	67	ND		3.7	
77	m	75	ND		0.6	
103	m	64	ND		36.2	
108	m	55	ND		6.7	
146	f	38	negative for IDH1 (R132H)		11.8	
162	m	58	negative for IDH1 (R132H)		19.9	
167	f	63	negative for IDH1 (R132H)		5	
196	m	50	negative for IDH1 (R132H)		3.4	
224	f	43	negative for IDH1 (R132H)		10.9	
233	m	66	negative for IDH1 (R132H)		39.1	
256	m	52	mutated IDH1 (R132H)		34.8	
275	m	60	negative for IDH1 (R132H)		17.7	
311	m	28	mutated IDH1 (R132H)		26.6	
363	m	40	negative for IDH1 (R132H)		7	
Median survival					18.8	
12	m	59	ND		86.9	4
34	m	62	ND		1.8	
49	m	75	ND		1.8	
59	m	65	ND		8.5	
109 recurrence of GBM54	f	41			26.1	
220	m	58	negative for IDH1 (R132H)		14.5	
228	m	83	negative for IDH1 (R132H)		0.3	
Median survival					20	
**B.**						
**No.**	**Sex**	**Age at Diagnosis**	**IDH1 Status**		**Survival (months)**	**Recurrence**
13	f	47	ND		20.9	
42	f	27	ND		17.4	
60	m	51	ND		42	
115	f	27	negative for IDH1 (R132H)		57.5	
173	m	46	negative for IDH1 (R132H)		7.8	
236	m	17	negative for IDH1 (R132H)		24.6	
287	f	52	mutated IDH1 (R132H)		30.5	
295	m	33	negative for IDH1 (R132H)		29.8	
302	m	36	mutated IDH1 (R132H)		28.4	
310	m	18	ND		24.6	recurrence of AS-236
337	m	65	negative for IDH1 (R132H)		17.5	
355	f	31	negative for IDH1 (R132H)		57.5	recurrence of AS-115
382	m	32	mutated IDH1 (R132H)		8.1	
Median survival					28.2	
**C.**						
**No.**	**Sex**	**Age at Diagnosis**	**IDH1 Status**	**Survival (Months)**	**WHO Grade**	**Recurrence**
22	f	36	ND	83	2	
32	m	35	ND	80.7	3	
37	f	63	ND	2.4	2	
62	m	27	ND	69.7	2	
83	m	39	ND	64.5	2	
134	m	32	mutated IDH1 (R132H)	102.3	3	Yes
152	m	28	negative for IDH1 (R132H)	69.7	3	Yes
158	f	41	negative for IDH1 (R132H)	51.2	2	
160	m	33	mutated IDH1 (R132H)	53.3	3	
172	m	55	mutated IDH1 (R132H)	18.9	3	
188	f	26	negative for mutated IDH1 (R132H)	46.4	2	
193	m	35	negative for mutated IDH1 (R132H)	45.2	3	
197	f	29	negative for mutated IDH1 (R132H)	45	2	
211	m	43	mutated IDH1 (R132H)	42.1	2	
218	m	31	mutated IDH1 (R132H)	41.4	2	
225	m	33	mutated IDH1 (R132H)	102.3	3	Yes
238	m	66	mutated IDH1 (R132H)	38.5	3	
242	m	23	mutated IDH1 (R132H)	67.3	2	Yes
325	m	30	mutated IDH1 (R132H)	23.7	2	
341	m	45	mutated IDH1 (R132H)	22.8	2	
372	m	28	mutated IDH1 (R132H)	10.6	3	
Median survival				51.5		

**Table 2:** Antibodies used for immunodetection.

Target Protein	Company and Catalog Number	Species	Dilution
CD59	CST, #65055	Rabbit monoclonal	1:1000
STAT1	CST, #9176	Mouse monoclonal	1:1000
STAT6	ABCAM, ab32520	Rabbit monoclonal	1:1000 Western blot; 1:50 IHC
Creatinine Kinase, M-Type (CKM)	ABCAM, ab151465	Rabbit polyclonal	1:1000
Fibronectin 1 (FN1)	ABCAM, ab2413	Rabbit polyclonal	1:1000 Western blot; 1:200 IF
Midkine (MDK)	ABCAM, ab52637	Rabbit monoclonal	1:1000 Western blot
Midkine (MDK)	ABCAM, ab170820	Rabbit polyclonal	1:50 IHC
β-actin	SCBT, sc47778	Mouse monoclonal	1:10,000
Biotinylated Goat anti Rabbit	Vector Laboratories, BA-1000		1:200
HRP conjugated Goat anti-Mouse	CST, 7076		1:2000
Alexa Fluor 594 conjugated Goat anti Rabbit	Thermo Fisher, A11012		1:1000
HRP conjugated Goat anti-Rabbit	CST, 7074		1:2000

**Table 3:** Primers used for qPCR analysis.

Target	Forward	Reverse
MDK	5′-CGCGGTCGCCAAAAAGAAAG-3′	5′-ACTTGCAGTCGGCTCCAAAC-3′
PTP	5′-GTGGAGACTGTGG GCTGGG-3′	5′-GCCTTCCTTTTTCTTCTTCTTAG-3′
PTPRZ1	5′-GTGTCAGCGGAGGAGTTTCAG-3′	5′-CTGCTTCCCAAAACGACTAACAC-3′
ALK1	5′-ACCGACTACAACCCCAACTAC-3′	5′-ACCCCAATGCAGCGAACAATG-3′
ALK2	5′-CTTCATCCACCGAGACATTGCT-3′	5′-GGGCAGTTCTTGGGTGGGTC-3′
NOTCH2	5′- CAGAAGATGTGGATGAATGTGC-3′	5′- GACTTTATCCACACACTGCCC -3′
Nucleolin	5′-GAAGGCACAGAACCGACTACG-3′	5′-CCTTTACTTTTCCCATCCTTGC-3′
SDC3	5′-CGATGATGAACTGGATGACCTC-3′	5′-ATGGTAGTGGAGACGGTGGTG-3′
SDC4	5′-CCAGACGATGAGGATGTAGTG-3′	5′-ACACATCCTCACTCTCTTCAAC-3′
LRP6	5′-GAGAAGTGCCAAAGATAGAACG-3′	5′-TTCACGCAGACCCTCACCAG-3′
LRP8	5′-CTACCCTGGCTACGAGATGG-3′	5′-CTCCTGCTCTTTCGGGTCAC-3′
CSPG5	5′-TCAGTGTGCGACCTCTTCCC-3′	5′-GGGAGAAGTTATCATTGTGGAG-3′
GAPDH	5′-GTCTCCTCTGACTTCAACAGCG-3′	5′-ACCACCCTGTTGCTGTAGCCAA-3′

## Data Availability

The data that support the findings of this study are available from the corresponding author upon reasonable request.

## Supplementary Materials

The following are available online at https://www.mdpi.com/article/10.3390/ijms22179566/s1.

## References

[1] Friedman H.S., Kerby T., Calvert H. (2000). Temozolomide and treatment of malignant glioma. Clin. Cancer Res..

[2] Krex D., Klink B., Hartmann C., von Deimling A., Pietsch T., Simon M., Sabel M., Steinbach J.P., Heese O., Reifenberger G. (2007). Long-term survival with glioblastoma multiforme. Brain.

[3] Stupp R., Tonn J.C., Brada M., Pentheroudakis G. (2010). High-grade malignant glioma: ESMO Clinical Practice Guidelines for diagnosis, treatment and follow-up. Ann. Oncol..

[4] Gurney J.G., Kadan-Lottick N. (2001). Brain and other central nervous system tumors: Rates, trends, and epidemiology. Curr. Opin. Oncol..

[5] Ohgaki H., Dessen P., Jourde B., Horstmann S., Nishikawa T., Di Patre P.L., Burkhard C., Schüler D., Probst-Hensch N.M., Maiorka P.C. (2004). Genetic pathways to glioblastoma: A population-based study. Cancer Res..

[6] Stupp R., Bent M.V.D., Hegi M. (2005). Optimal role of temozolomide in the treatment of malignant gliomas. Curr. Neurol. Neurosci. Rep..

[7] Stupp R., Taillibert S., Kanner A., Read W., Steinberg D., Lhermitte B., Toms S., Idbaih A., Ahluwalia M.S., Fink K. (2017). Effect of Tumor-Treating Fields Plus Maintenance Temozolomide vs Maintenance Temozolomide Alone on Survival in Patients with Glioblastoma: A Randomized Clinical Trial. JAMA.

[8] Mrugala M.M., Chamberlain M.C. (2008). Mechanisms of Disease: Temozolomide and glioblastoma—look to the future. Nat. Clin. Pract. Oncol..

[9] Phillips H.S., Kharbanda S., Chen R., Forrest W.F., Soriano R.H., Wu T.D., Misra A., Nigro J.M., Colman H., Soroceanu L. (2006). Molecular subclasses of high-grade glioma predict prognosis, delineate a pattern of disease progression, and resemble stages in neurogenesis. Cancer Cell.

[10] Shen Y., Grisdale C., Islam S.A., Bose P., Lever J., Zhao E.Y., Grinshtein N., Ma Y., Mungall A.J., Moore R.A. (2019). Comprehensive genomic profiling of glioblastoma tumors, BTICs, and xenografts reveals stability and adaptation to growth environments. Proc. Natl. Acad. Sci. USA.

[11] Verhaak R.G., Hoadley K.A., Purdom E., Wang V., Qi Y., Wilkerson M.D., Miller C.R., Ding L., Golub T., Mesirov J.P. (2010). Integrated genomic analysis identifies clinically relevant subtypes of glioblastoma characterized by abnormalities in PDGFRA, IDH1, EGFR, and NF1. Cancer Cell.

[12] Dekker L.J.M., Kannegieter N.M., Haerkens F., Toth E., Kros J.M., Hov D.A.S., Fillebeen J., Verschuren L., Leenstra S., Ressa A. (2020). Multiomics profiling of paired primary and recurrent glioblastoma patient tissues. Neuro-Oncol. Adv..

[13] Daubon T., Guyon J., Raymond A.-A., Dartigues B., Rudewicz J., Ezzoukhry Z., Dupuy J.-W., Herbert J.M.J., Saltel F., Bjerkvig R. (2019). The invasive proteome of glioblastoma revealed by laser-capture microdissection. Neuro-Oncol. Adv..

[14] De Aquino P.F., Carvalho P.C., Nogueira F.C., da Fonseca C.O., de Souza Silva J.C., da Costa Carvalho M.d.G., Domont G.B., Zanchin N.I., da Gama Fischer S.F. (2016). A Time-Based and Intratumoral Proteomic Assessment of a Recurrent Glioblastoma Multiforme. Front. Oncol..

[15] Polisetty R.V., Gupta M.K., Nair S.C., Ramamoorthy K., Tiwary S., Shiras A., Chandak G.R., Sirdeshmukh R. (2011). Glioblastoma cell secretome: Analysis of three glioblastoma cell lines reveal 148 non-redundant proteins. J. Proteom..

[16] Okawa S., Gagrica S., Blin C., Ender C., Pollard S.M., Krijgsveld J. (2017). Proteome and Secretome Characterization of Glioblastoma-Derived Neural Stem Cells. Stem Cells.

[17] Tian Q., Sangar V., Price N.D. (2016). Emerging Proteomic Technologies Provide Enormous and Underutilized Potential for Brain Cancer Research. Mol. Cell. Proteom..

[18] Wang L.-B., Karpova A., Gritsenko M.A., Kyle J.E., Cao S., Li Y., Rykunov D., Colaprico A., Rothstein J.H., Hong R. (2021). Proteogenomic and metabolomic characterization of human glioblastoma. Cancer Cell.

[19] Karsy M., Neil J.A., Guan J., Mahan M.A., Colman H., Jensen R.L. (2015). A practical review of prognostic correlations of molecular biomarkers in glioblastoma. Neurosurg. Focus.

[20] Wick W., Weller M., Bent M.V.D., Sanson M., Weiler M., Von Deimling A., Plass C., Hegi M., Platten M., Reifenberger G. (2014). MGMT testing-the challenges for biomarker-based glioma treatment. Nat. Rev. Neurol..

[21] Huse J.T., Aldape K.D. (2014). The Evolving Role of Molecular Markers in the Diagnosis and Management of Diffuse Glioma. Clin. Cancer Res..

[22] Sreekanthreddy P., Srinivasan H., Kumar D.M., Nijaguna M.B., Sridevi S., Vrinda M., Arivazhagan A., Balasubramaniam A., Hegde A.S., Chandramouli B.A. (2010). Identification of Potential Serum Biomarkers of Glioblastoma: Serum Osteopontin Levels Correlate with Poor Prognosis. Cancer Epidemiol. Biomark. Prev..

[23] Gold L., Ayers D., Bertino J., Bock C., Bock A., Brody E.N., Carter J., Dalby A.B., Eaton B.E., Fitzwater T. (2010). Aptamer-based multiplexed proteomic technology for biomarker discovery. PLoS ONE.

[24] Gold L., Walker J.J., Wilcox S.K., Williams S. (2012). Advances in human proteomics at high scale with the SOMAscan proteomics platform. New Biotechnol..

[25] Mehan M.R., Ayers D., Thirstrup D., Xiong W., Ostroff R.M., Brody E.N., Walker J.J., Gold L., Jarvis T.C., Janjic N. (2012). Protein Signature of Lung Cancer Tissues. PLoS ONE.

[26] Graumann J., Finkernagel F., Reinartz S., Stief T., Brödje D., Renz H., Jansen J.M., Wagner U., Worzfeld T., Von Strandmann E.P. (2019). Multi-platform Affinity Proteomics Identify Proteins Linked to Metastasis and Immune Suppression in Ovarian Cancer Plasma. Front. Oncol..

[27] Mysona D., Pyrzak A., Purohit S., Zhi W., Sharma A., Tran L., Tran P., Bai S., Rungruang B., Ghamande S. (2019). A combined score of clinical factors and serum proteins can predict time to recurrence in high grade serous ovarian cancer. Gynecol. Oncol..

[28] Tsim S., Kelly C., Alexander L., McCormick C., Thomson F., Woodward R., Foster J.E., Stobo D.B., Paul J., Maskell N.A. (2016). Diagnostic and Prognostic Biomarkers in the Rational Assessment of Mesothelioma (DIAPHRAGM) study: Protocol of a prospective, multicentre, observational study. BMJ Open.

[29] Ostroff R.M., Mehan M.R., Stewart A., Ayers D., Brody E.N., Williams S.A., Levin S., Black B., Harbut M., Carbone M. (2012). Early Detection of Malignant Pleural Mesothelioma in Asbestos-Exposed Individuals with a Noninvasive Proteomics-Based Surveillance Tool. PLoS ONE.

[30] Qiao Z., Pan X., Parlayan C., Ojima H., Kondo T. (2017). Proteomic study of hepatocellular carcinoma using a novel modified aptamer-based array (SOMAscan) platform. Biochim. Biophys. Acta Proteins Proteom..

[31] Brody E., Gold L., Mehan M., Ostroff R., Rohloff J., Walker J., Zichi D. (2012). Life’s simple measures: Unlocking the proteome. J. Mol. Biol..

[32] Gold L., Janjic N., Jarvis T., Schneider D., Walker J.J., Wilcox S.K., Zichi D. (2010). Aptamers and the RNA World, Past and Present. Cold Spring Harb. Perspect. Biol..

[33] Cao K.-A.L., Boitard S., Besse P. (2011). Sparse PLS discriminant analysis: Biologically relevant feature selection and graphical displays for multiclass problems. BMC Bioinform..

[34] Minniti G., Scaringi C., Arcella A., Lanzetta G., Di Stefano D., Scarpino S., Bozzao A., Pace A., Villani V., Salvati M. (2014). IDH1 mutation and MGMT methylation status predict survival in patients with anaplastic astrocytoma treated with temozolomide-based chemoradiotherapy. J. Neuro-Oncol..

[35] Christians A., Adel-Horowski A., Banan R., Lehmann U., Bartels S., Behling F., Barrantes-Freer A., Stadelmann C., Rohde V., Stockhammer F. (2019). The prognostic role of IDH mutations in homogeneously treated patients with anaplastic astrocytomas and glioblastomas. Acta Neuropathol. Commun..

[36] Xia L., Wu B., Fu Z., Feng F., Qiao E., Li Q., Sun C., Ge M. (2015). Prognostic role of IDH mutations in gliomas: A meta-analysis of 55 observational studies. Oncotarget.

[37] Gadji M., Fortin D., Tsanaclis A.-M., Drouin R. (2009). Is the 1p/19q deletion a diagnostic marker of oligodendrogliomas?. Cancer Genet. Cytogenet..

[38] Civita P., Franceschi S., Aretini P., Ortenzi V., Menicagli M., Lessi F., Pasqualetti F., Naccarato A.G., Mazzanti C.M. (2019). Laser Capture Microdissection and RNA-Seq Analysis: High Sensitivity Approaches to Explain Histopathological Heterogeneity in Human Glioblastoma FFPE Archived Tissues. Front. Oncol..

[39] Dirkse A., Golebiewska A., Buder T., Nazarov P.V., Muller A., Poovathingal S.K., Brons N.H.C., Leite S., Sauvageot N., Sarkisjan D. (2019). Stem cell-associated heterogeneity in Glioblastoma results from intrinsic tumor plasticity shaped by the microenvironment. Nat. Commun..

[40] Schäfer N., Gielen G.H., Rauschenbach L., Kebir S., Till A., Reinartz R., Simon M., Niehusmann P., Kleinschnitz C., Herrlinger U. (2019). Longitudinal heterogeneity in glioblastoma: Moving targets in recurrent versus primary tumors. J. Transl. Med..

[41] Shi L., Westwood S., Baird A.L., Winchester L., Dobricic V., Kilpert F., Hong S., Franke A., Hye A., Ashton N.J. (2019). Discovery and validation of plasma proteomic biomarkers relating to brain amyloid burden by SOMAscan assay. Alzheimer’s Dement..

[42] Muramatsu T. (2014). Structure and function of midkine as the basis of its pharmacological effects. Br. J. Pharmacol..

[43] Jono H., Ando Y. (2010). Midkine: A Novel Prognostic Biomarker for Cancer. Cancers.

[44] Kato S., Ishihara K., Shinozawa T., Yamaguchi H., Asano Y., Saito M., Kato M., Terada T., Awaya A., Hirano A. (1999). Monoclonal antibody to human midkine reveals increased midkine expression in human brain tumors. J. Neuropathol. Exp. Neurol..

[45] Mishima K., Asai A., Kadomatsu K., Ino Y., Nomura K., Narita Y., Muramatsu T., Kirino T. (1997). Increased expression of midkine during the progression of human astrocytomas. Neurosci. Lett..

[46] Luo J., Wang X., Xia Z., Yang L., Ding Z., Chen S., Lai B., Zhang N. (2015). Transcriptional factor specificity protein 1 (SP1) promotes the proliferation of glioma cells by up-regulating midkine (MDK). Mol. Biol. Cell.

[47] Kishida S., Mu P., Miyakawa S., Fujiwara M., Abe T., Sakamoto K., Onishi A., Nakamura Y., Kadomatsu K. (2013). Midkine Promotes Neuroblastoma through Notch2 Signaling. Cancer Res..

[48] Herradon G., Ramos-Alvarez M.P., Gramage E. (2019). Connecting Metainflammation and Neuroinflammation Through the PTN-MK-RPTPbeta/zeta Axis: Relevance in Therapeutic Development. Front. Pharm..

[49] Wang J., Takeuchi H., Sonobe Y., Jin S., Mizuno T., Miyakawa S., Fujiwara M., Nakamura Y., Kato T., Muramatsu H. (2008). Inhibition of midkine alleviates experimental autoimmune encephalomyelitis through the expansion of regulatory T cell population. Proc. Natl. Acad. Sci. USA.

[50] Hao H., Maeda Y., Fukazawa T., Yamatsuji T., Takaoka M., Bao X.H., Matsuoka J., Okui T., Shimo T., Takigawa N. (2013). Inhibition of the growth factor MDK/midkine by a novel small molecule compound to treat non-small cell lung cancer. PLoS ONE.

[51] Huang H.-L., Shen J.-F., Min L.-S., Ping J.-L., Lu Y.-L., Dai L.-C. (2015). Inhibitory effect of midkine-binding peptide on tumor proliferation and migration. Int. J. Clin. Exp. Pathol..

[52] Ma J., Lang B., Wang X., Wang L., Dong Y., Hu H. (2014). Co-expression of midkine and pleiotrophin predicts poor survival in human glioma. J. Clin. Neurosci..

[53] Toledano-Katchalski H., Nir R., Volohonsky G., Volk T. (2007). Post-transcriptional repression of the Drosophila midkine and pleiotrophin homolog miple by HOW is essential for correct mesoderm spreading. Development.

[54] Suzuki N., Shibata Y., Urano T., Murohara T., Muramatsu T., Kadomatsu K. (2004). Proteasomal Degradation of the Nuclear Targeting Growth Factor Midkine. J. Biol. Chem..

[55] Zhang D., Ding L., Li Y., Ren J., Shi G., Wang Y., Zhao S., Ni Y., Hou Y. (2017). Midkine derived from cancer-associated fibroblasts promotes cisplatin-resistance via up-regulation of the expression of lncRNA ANRIL in tumour cells. Sci. Rep..

[56] Rawnaq T., Kunkel M., Simon R., Zander H., Bachmann K., Sauter G., Izbicki J.R., Kaifi J.T. (2011). Serum midkine correlates with tumor progression and imatinib response in gastrointestinal stromal tumors. Ann. Surg. Oncol..

[57] Sun B., Hu C., Yang Z., Zhang X., Zhao L., Xiong J., Ma J., Chen L., Qian H., Luo X. (2017). Midkine promotes hepatocellular carcinoma metastasis by elevating anoikis resistance of circulating tumor cells. Oncotarget.

[58] Xu Y.-Y., Mao X.-Y., Song Y.-X., Zhao F., Wang Z.-N., Zhang W.-X., Xu H.-M., Jin F. (2012). Midkine confers Adriamycin resistance in human gastric cancer cells. Tumor Biol..

[59] Zeller C., Dai W., Steele N.L., Siddiq A., Walley A., Wilhelm-Benartzi C., Rizzo S., Van Der Zee A., Plumb J.A., Brown R. (2012). Candidate DNA methylation drivers of acquired cisplatin resistance in ovarian cancer identified by methylome and expression profiling. Oncogene.

[60] Kadomatsu K., Muramats T. (2004). Midkine and pleiotrophin in neural development and cancer. Cancer Lett..

[61] Muramatsu H., Zou P., Suzuki H., Oda Y., Chen G.Y., Sakaguchi N., Sakuma S., Maeda N., Noda M., Takada Y. (2004). alpha4beta1- and alpha6beta1-integrins are functional receptors for midkine, a heparin-binding growth factor. J. Cell Sci..

[62] Mitsiadis T.A., Salmivirta M., Muramatsu T., Muramatsu H., Rauvala H., Lehtonen E., Jalkanen M., Thesleff I. (1995). Expression of the heparin-binding cytokines, midkine (MK) and HB-GAM (pleiotrophin) is associated with epithelial-mesenchymal interactions during fetal development and organogenesis. Development.

[63] Kojima T., Katsumi A., Yamazaki T., Muramatsu T., Nagasaka T., Ohsumi K., Saito H. (1996). Human ryudocan from endothelium-like cells binds basic fibroblast growth factor, midkine, and tissue factor pathway inhibitor. J. Biol. Chem..

[64] Nakanishi T., Kadomatsu K., Okamoto T., Ichihara-Tanaka K., Kojima T., Saito H., Tomoda Y., Muramatsu T. (1997). Expression of syndecan-1 and -3 during embryogenesis of the central nervous system in relation to binding with midkine. J. Biochem..

[65] XGuo X., Pan Y., Xiong M., Sanapala S., Anastasaki C., Cobb O., Dahiya S., Gutmann D.H. (2020). Midkine activation of CD8+ T cells establishes a neuron–immune–cancer axis responsible for low-grade glioma growth. Nat. Commun..

[66] Hemmer W., Wallimann T. (1993). Functional Aspects of Creatine Kinase in Brain. Dev. Neuroscience.

[67] Róna E., Nagy A., Wollemann M., Slowik F. (1972). Localization of various isoenzymes in different cell fractions of brain tumours. Neuropatol. Pol..

[68] Tsung S.H. (1983). Creatine kinase activity and isoenzyme pattern in various normal tissues and neoplasms. Clin. Chem..

[69] Wallimann T., Tokarska-Schlattner M., Schlattner U. (2011). The creatine kinase system and pleiotropic effects of creatine. Amino Acids.

[70] Andres R.H., Wallimann T., Widmer H.R. (2016). Creatine supplementation improves neural progenitor cell survival in Huntington’s disease. Brain Circ..

[71] Gramage E., Herradon G., Martin Y.B., Vicente-Rodriguez M., Rojo L., Gnekow H., Barbero A., Perez-Garcia C. (2013). Differential phosphoproteome of the striatum from pleiotrophin knockout and midkine knockout mice treated with amphetamine: Correlations with amphetamine-induced neurotoxicity. Toxicology.

[72] Mäenpää A., Junnikkala S., Hakulinen J., Timonen T., Meri S. (1996). Expression of complement membrane regulators membrane cofactor protein (CD46), decay accelerating factor (CD55), and protectin (CD59) in human malignant gliomas. Am. J. Pathol..

[73] Marx S., Wilken F., Wagner I., Marx M., Troschke-Meurer S., Zumpe M., Bien-Moeller S., Weidemeier M., Baldauf J., Fleck S.K. (2020). GD2 targeting by dinutuximab beta is a promising immunotherapeutic approach against malignant glioma. J. Neuro Oncol..

[74] Sonmez H.A., Kökoǧlu E., Süer S., Özyurt E. (1995). Fibronectin and sialic acid levels in human meningiomas and gliomas. Cancer Lett..

[75] Kochi N., Tani E., Morimura T., Itagaki T. (1983). Immunohistochemical study of fibronectin in human glioma and meningioma. Acta Neuropathol..

[76] Paetau A. (1988). Glial fibrillary acidic protein, vimentin and fibronectin in primary cultures of human glioma and fetal brain. Acta Neuropathol..

[77] Yang C.H., Wang Y., Sims M., Cai C., Pfeffer L.M. (2019). MicroRNA-1 suppresses glioblastoma in preclinical models by targeting fibronectin. Cancer Lett..

[78] Sengupta S., Nandi S., Hindi E.S., Wainwright D.A., Han Y., Lesniak M.S. (2010). Short hairpin RNA-mediated fibronectin knockdown delays tumor growth in a mouse glioma model. Neoplasia.

[79] Wang J.P., Hielscher A. (2017). Fibronectin: How Its Aberrant Expression in Tumors May Improve Therapeutic Targeting. J. Cancer.

[80] Daniels D.A., Chen H., Hicke B.J., Swiderek K.M., Gold L. (2003). A tenascin-C aptamer identified by tumor cell SELEX: Systematic evolution of ligands by exponential enrichment. Proc. Natl. Acad. Sci. USA.

[81] Chung D., Keles S. (2010). Sparse Partial Least Squares Classification for High Dimensional Data. Stat. Appl. Genet. Mol. Biol..

[82] Rohart F., Gautier B., Singh A., Le Cao K.-A. (2017). mixOmics: An R package for ‘omics feature selection and multiple data integration. PLoS Comput. Biol..

[83] Shannon P., Markiel A., Ozier O., Baliga N.S., Wang J.T., Ramage D., Amin N., Schwikowski B., Ideker T. (2003). Cytoscape: A Software Environment for Integrated Models of Biomolecular Interaction Networks. Genome Res..

[84] Bindea G., Mlecnik B., Hackl H., Charoentong P., Tosolini M., Kirilovsky A., Fridman W.-H., Pagès F., Trajanoski Z., Galon J. (2009). ClueGO: A Cytoscape plug-in to decipher functionally grouped gene ontology and pathway annotation networks. Bioinformatics.

