# Supplementary material for: Slow Off-Rate Modified Aptamer (SOMAmer) Proteomic Analysis of Patient-Derived Malignant Glioma Identifies Distinct Cellular Proteomes
Source: Int J Mol Sci. 2021 Sep 3;22(17):9566. doi: 10.3390/ijms22179566 (PMC8431317; doi:10.3390/ijms22179566)
Supplement: Supplementary file 1 [file ijms-22-09566-s001.zip › ijms-1334162-supplementary.pptx]

## Slide 1
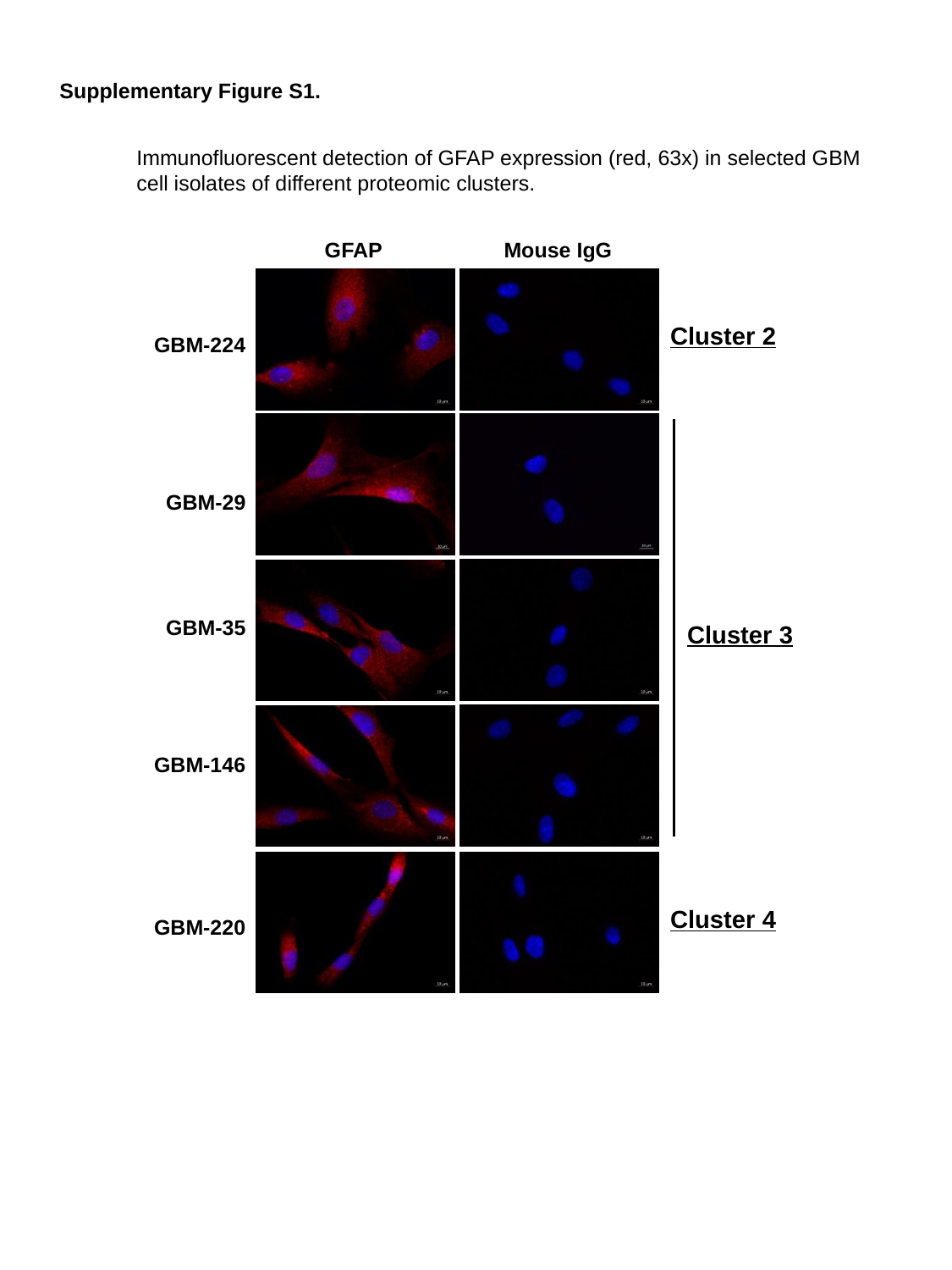

Supplementary Figure S1.
Immunofluorescent detection of GFAP expression (red, 63x) in selected GBM cell isolates of different proteomic clusters.
Mouse IgG
GFAP
Cluster 2
GBM-224
GBM-29
GBM-35
Cluster 3
GBM-146
Cluster 4
GBM-220

## Slide 2
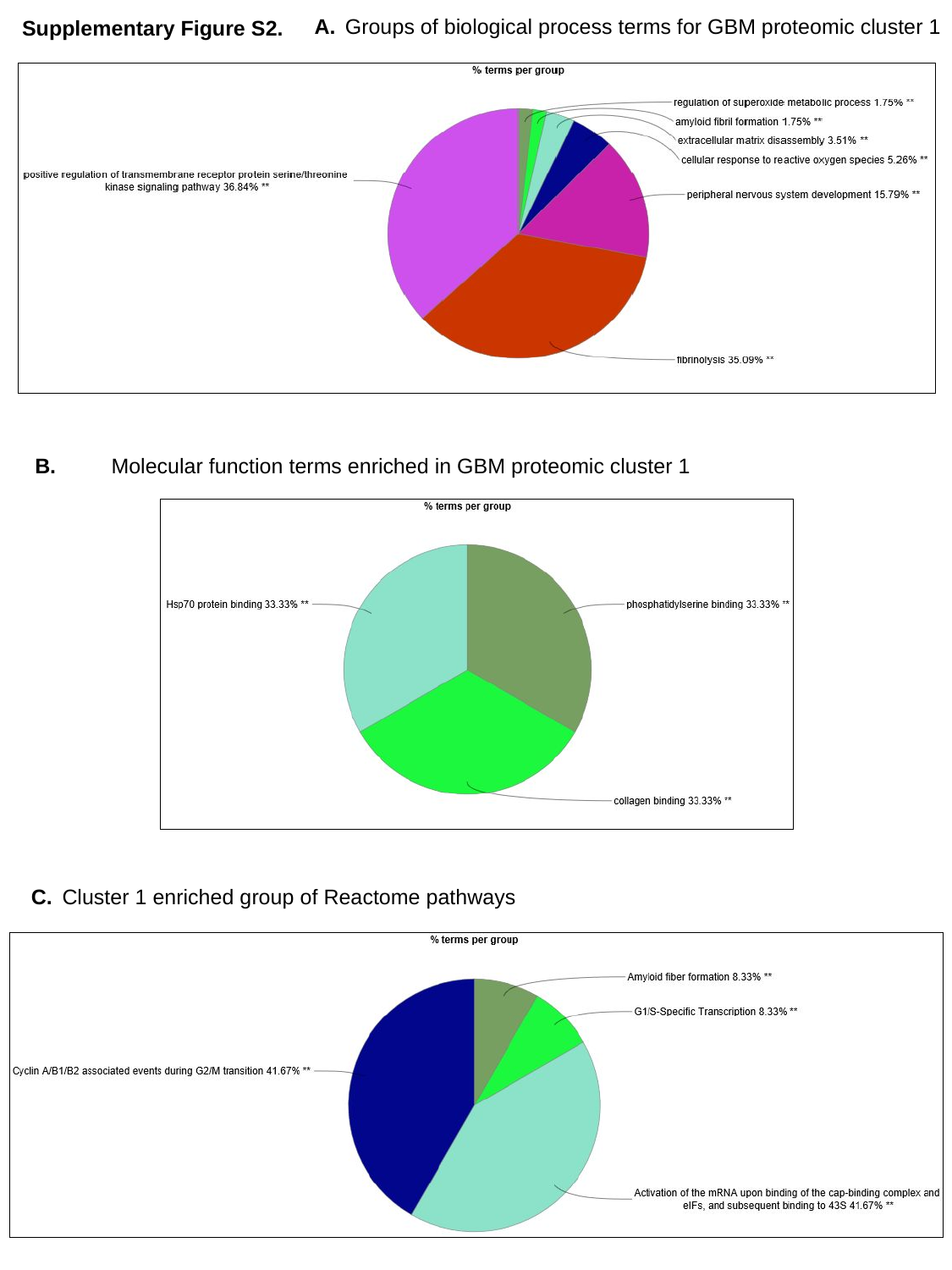

A.
Groups of biological process terms for GBM proteomic cluster 1
Supplementary Figure S2.
B.
Molecular function terms enriched in GBM proteomic cluster 1
Cluster 1 enriched group of Reactome pathways
C.

## Slide 3
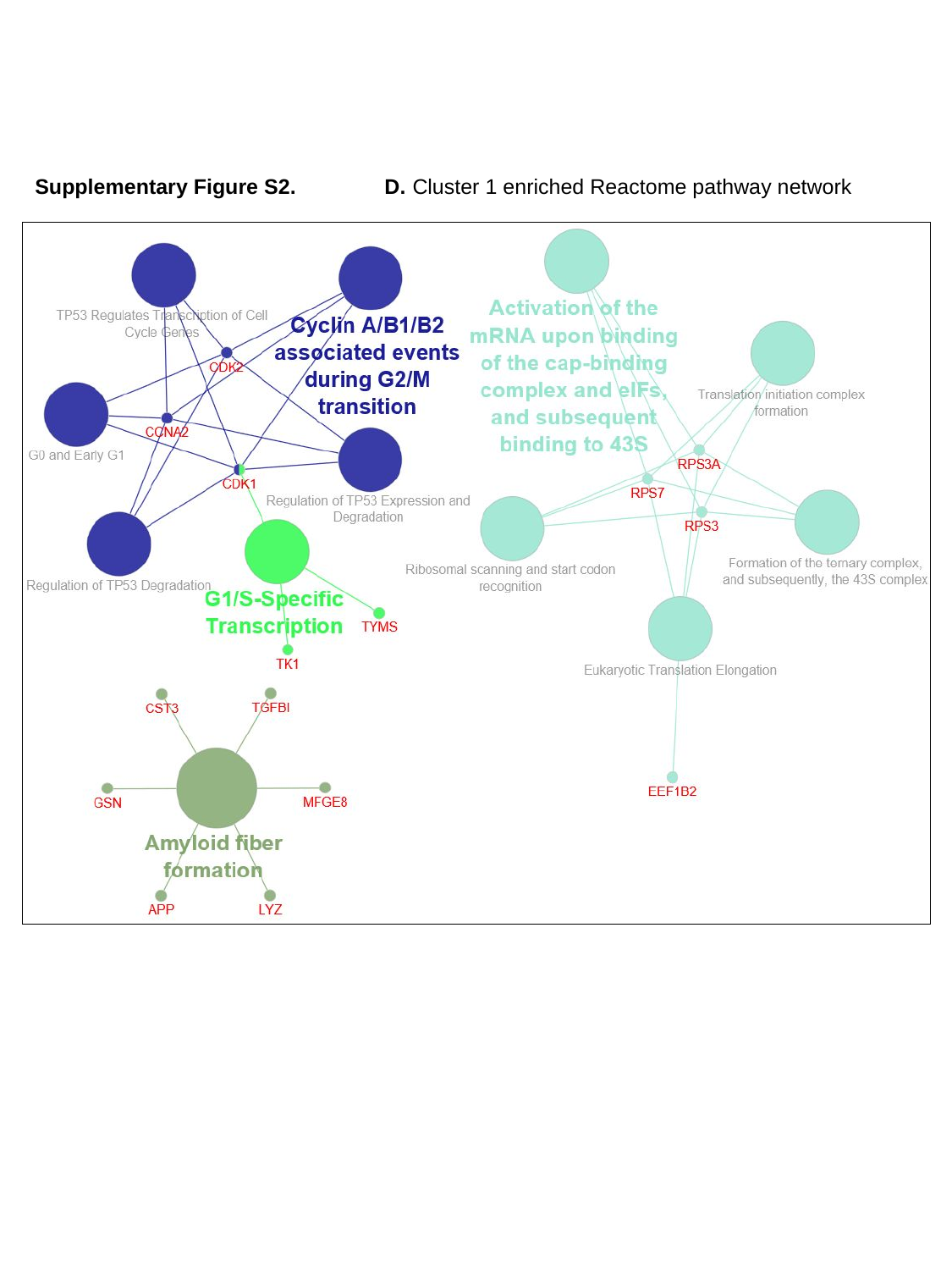

Supplementary Figure S2. D. Cluster 1 enriched Reactome pathway network

## Slide 4
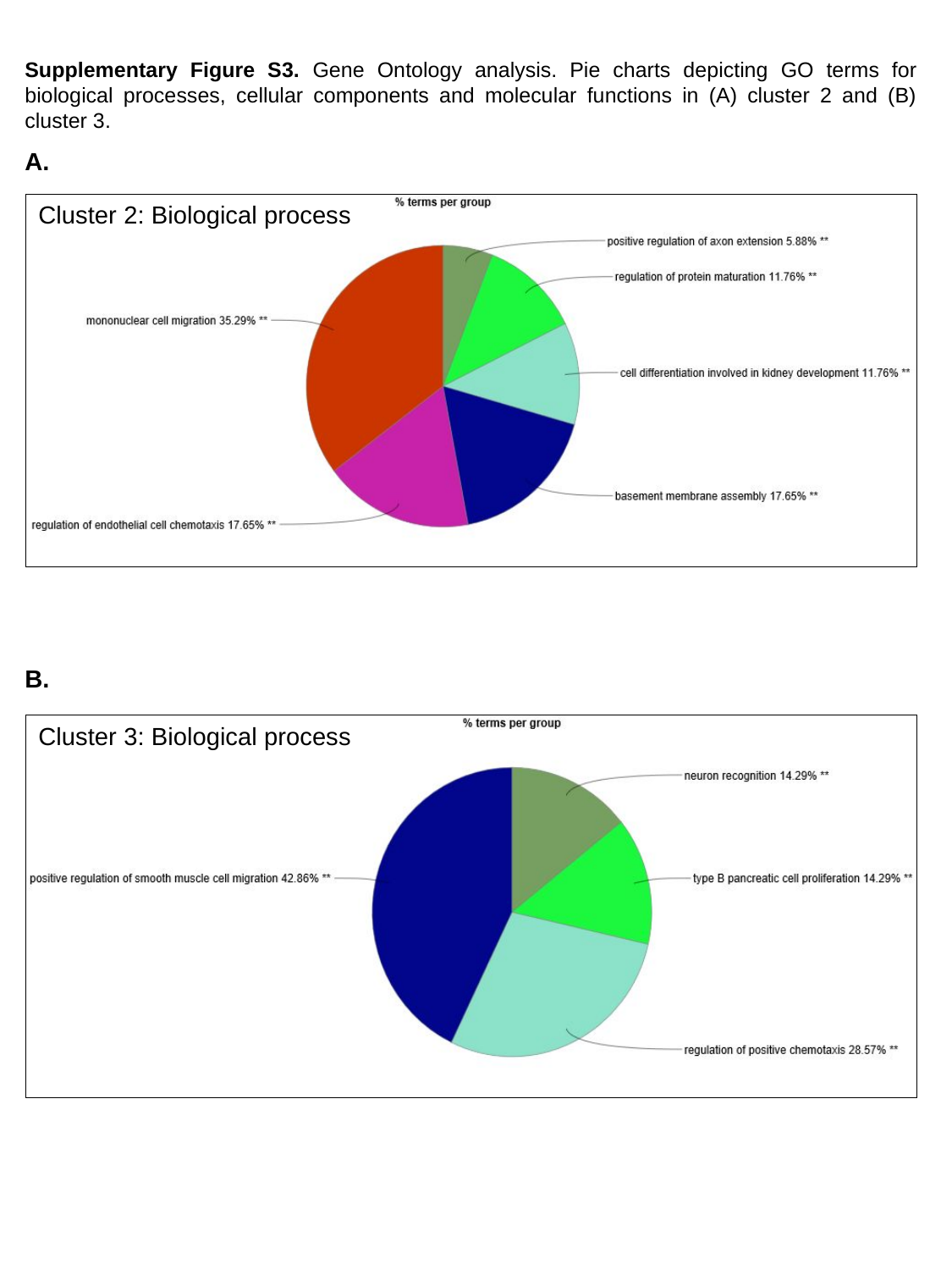

Supplementary Figure S3. Gene Ontology analysis. Pie charts depicting GO terms for biological processes, cellular components and molecular functions in (A) cluster 2 and (B) cluster 3.
A.
Cluster 2: Biological process
B.
Cluster 3: Biological process

## Slide 5
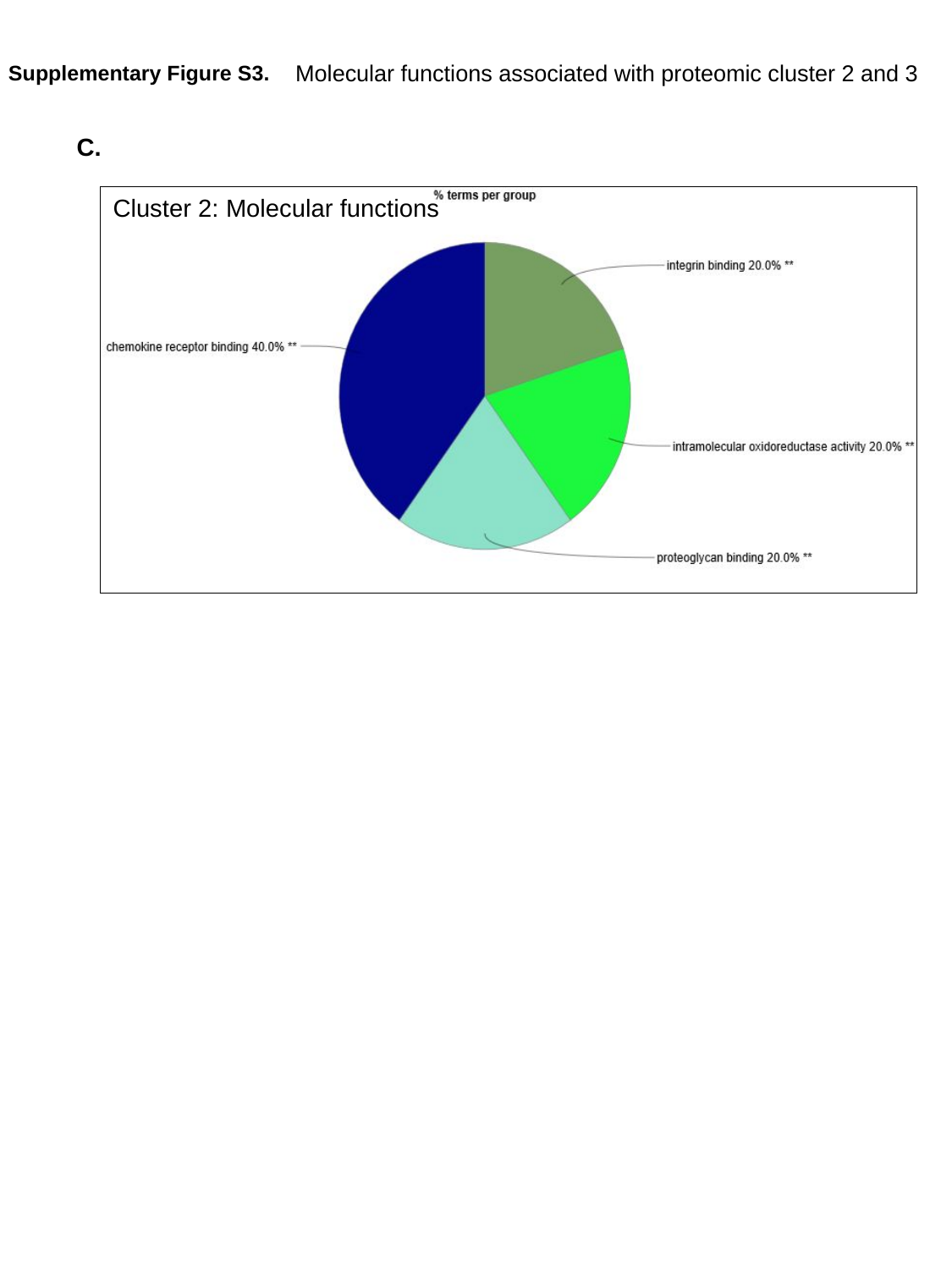

Molecular functions associated with proteomic cluster 2 and 3
Supplementary Figure S3.
C.
Cluster 2: Molecular functions
